# New role of P2X7 receptor in an Alzheimer’s disease mouse model

**DOI:** 10.1038/s41380-018-0108-3

**Published:** 2018-06-22

**Authors:** Elodie Martin, Majid Amar, Carine Dalle, Ihsen Youssef, Céline Boucher, Caroline Le Duigou, Matthias Brückner, Annick Prigent, Véronique Sazdovitch, Annett Halle, Jean M. Kanellopoulos, Bertrand Fontaine, Benoît Delatour, Cécile Delarasse

**Affiliations:** 1 Inserm, CNRS, Sorbonne Universités, Institut du Cerveau et de la Moelle épinière, ICM, F-75013 Paris, France; 2 Center of Advanced European Studies and Research (caesar), Max Planck research group Neuroimmunology, 53175 Bonn, Germany; 3 AP-HP, Hôpital de la Pitié Salpêtrière, F-75013 Paris, France; 4 Institut de Biologie Intégrative, I2BC-CNRS 9198, Department of Biochemistry Biophysics and Structural Biology, Université Paris-Sud, 91405 Orsay, France; 5 Present Address: German Center for Neurodegenerative Diseases, 53127 Bonn, Germany

**Keywords:** Neuroscience, Physiology, Diseases

## Abstract

Extracellular aggregates of amyloid β (Aβ) peptides, which are characteristic of Alzheimer’s disease (AD), act as an essential trigger for glial cell activation and the release of ATP, leading to the stimulation of purinergic receptors, especially the P2X7 receptor (P2X7R). However, the involvement of P2X7R in the development of AD is still ill-defined regarding the dual properties of this receptor. Particularly, P2X7R activates the NLRP3 inflammasome leading to the release of the pro-inflammatory cytokine, IL-1β; however, P2X7R also induces cleavage of the amyloid precursor protein generating Aβ peptides or the neuroprotective fragment sAPPα. We thus explored in detail the functions of P2X7R in AD transgenic mice. Here, we show that P2X7R deficiency reduced Aβ lesions, rescued cognitive deficits and improved synaptic plasticity in AD mice. However, the lack of P2X7R did not significantly affect the release of IL-1β or the levels of non-amyloidogenic fragment, sAPPα, in AD mice. Instead, our results show that P2X7R plays a critical role in Aβ peptide-mediated release of chemokines, particularly CCL3, which is associated with pathogenic CD8^+^ T cell recruitment. In conclusion, our study highlights a novel detrimental function of P2X7R in chemokine release and supports the notion that P2X7R may be a promising therapeutic target for AD.

## Introduction

Alzheimer’s disease (AD) is a neurodegenerative disease characterized by the formation of neurofibrillary tangles, composed of intraneuronal aggregates of hyperphosphorylated tau protein, and senile plaques, composed of extracellular aggregates of amyloid β (Aβ) peptides. These neuropathological accumulations are accompanied by a neuroinflammatory response [1, 2]. Moreover, genetic studies have identified several polymorphisms in genes involved in the innate immune response associated with AD [3–6]. Activated microglia and astrocytes surround Aβ lesions, and we recently showed that the immune response in murine cerebral amyloidosis models was characterized by the continuous production of several pro-inflammatory cytokines [7].

Furthermore, damaged neurons [8, 9], as well as microglia and astrocytes can release ATP in response to extracellular particles, such as Aβ peptides [10–13]. High extracellular ATP levels act as a danger signal, which activates purinergic receptors [14]. Purinergic receptors have various properties in the central nervous system; they contribute not only to neurotransmission and neuromodulation but also to neuronal injury depending on the physiological or pathological environment, the ATP concentration, the purinergic receptor subtype and the cell type that harbors these receptors [9, 15].

Among purinergic receptors, P2X7 receptor (P2X7R) may play a key role in the development and progression of AD [16]. P2X7R was reported to be upregulated near Aβ plaques in animal models of AD and in patients with AD [17, 18]. Moreover, P2X7R plays a role in activating the NLRP3 inflammasome, which is known to contribute to the pathogenic inflammatory response that occurs during AD. Indeed, in vitro Aβ stimulation of microglia caused activation of NLRP3, which induced IL-1β secretion [19]. Additionally, in an AD transgenic mouse model, NLRP3 inflammasome deficiency reversed the pro-inflammatory activation of microglia and improved disease progression [20]. Notably, P2X7R was shown to be involved in the Aβ-triggered release of IL-1β from microglia [10, 21], suggesting that P2X7R contributes to pro-inflammatory processes in AD. Additional reports indicated that early in vivo pharmacological blockade of P2X7R signaling had a protective effect by decreasing the Aβ load in AD mice (J20 model) [22].

Despite evidence that supports a role for P2X7R in AD pathogenesis, this receptor may also have neuroprotective effects [9]. For example, we previously demonstrated that brief P2X7R stimulation activated the beneficial, non-amyloidogenic processing of amyloid precursor protein (APP) [23, 24]. In this pathway, an α-secretase cleaves APP within the Aβ peptide sequence, which precludes the formation of neurotoxic Aβ peptides, and produces sAPPα, a neurotrophic and neuroprotective fragment [25]. Furthermore, a role for P2X7R in the phagocytosis of Aβ peptides was also reported to contribute to Aβ clearance. Thus, P2X7R might also down-regulate pathological microglial activation in AD [26].

Actually, the effects of P2X7R inhibition on cognitive deficits during AD stay an unanswered question and how P2X7R-dependent mechanisms participate in AD are ultimately still poorly understood, which stress the need for further in-depth study of the role of P2X7R in AD.

In the present study, we evaluated how the lack of P2X7R expression impacted a well-characterized mouse model of AD that develops Aβ brain lesions (APPPS1 mice) [27]. We examined, in detail, the potential role of P2X7R in AD pathological processes: memory and synaptic impairment, neuropathological alterations, APP processing, and immune response. We found that P2X7R deficiency decreased Aβ load and also rescued cognitive deficits in APPPS1 mice. Furthermore, we revealed a novel function of P2X7R in chemokine release associated with T-cells recruitment in Aβ pathology.

## Materials and methods

### Study approval

Post-mortem samples were obtained from a brain donation program of the French National Brain Bank, GIE NeuroCEB. All patients or the next of kin provided informed signed consent. Consent forms were reviewed and accepted by the Ethics Committee “Comité de Protection des Personnes Paris Ile de France VI”. The collection was declared to the Ministry of Research and Higher Education, and the Brain Bank was officially authorized to provide samples to project scientists (agreement AC-2013-1887). The project was approved by the Scientific Committee of the Brain Bank.

Mice were treated in accordance with the ARRIVE guidelines for the care and use of experimental animals of the European Union. C. D. and B. D. received official approval from the French Ministry of Agriculture to carry out research and experiments on animals (authorization no. A-75-1711 (C. D.), A-75-1741 (B. D.)). All procedures were approved by the Regional Ethics Committee and the French Ministry of Research and Higher Education (no. 01118.02).

### Human brain samples

A total of nine post-mortem brain samples (frontal cortex) were obtained; five from individuals with clinically and neuropathologically diagnosed AD (Braak stages: V–VI, Thal phases: 5) and four from non-demented controls (Braak stages: 0–IV, Thal phases: 0). Samples were fixed in 10% formalin solution and embedded in paraffin. Brain sections (5-µm thick) were cut on a microtome.

### Human tissue immunostaining

Human brain tissue sections were deparaffinized and pretreated with an antigen retrieval procedure (5 min in citrate buffer at 100 °C in a decloaking chamber) to unmask hidden epitopes. Then, tissue sections were incubated with a goat polyclonal anti-P2X7R antibody (Novus Biologicals, 1/100 dilution) overnight at 4 °C. Sections were then incubated with a secondary biotinylated rabbit anti-goat IgG for 30 min (Vector Labs, 1/250 dilution). Binding was detected with the peroxidase–avidin–biotin technique (ABC Elite Kit, Vector Labs) and 3,3′-diaminobenzidine (DAB, Sigma-Aldrich) as the chromogen. In each case, the digitized cortical sample was segmented, based on a fixed [160:210] threshold (ImageJ). Areas that displayed a signal above the threshold were summed and expressed as a percentage of the overall surface area of the cortical ribbon to calculate the P2X7R-labeled area.

For double immunostaining, human brain sections were incubated with primary antibodies against P2X7R (Novus Biologicals, 1/100 dilution), Aβ (Clone 4G8, mouse monoclonal IgG2b, 1/1000 dilution), Iba1 (rabbit polyclonal Ig fraction, 1/1000 dilution, Wako Chemicals USA, Inc.), and GFAP (rabbit polyclonal Ig fraction, 1/1000 dilution; Dako) overnight at 4 °C. Subsequently, samples were incubated with the appropriate Alexa Fluor secondary antibodies (1/1000 dilution, Life Technologies), for 2 h at room temperature. Brain sections were mounted with an aqueous medium (Fluoromount), and images were acquired on the Apotome.2 system for fluorescence microscopy (Zeiss).

### Animals

P2X7R knock-out (P2X7Rko) mice generated by Gabel’s group at Pfizer were obtained from Jackson Laboratory (number 005576) [28]. APPPS1 mice were obtained from the Jucker lab [27]. Both mouse lines had a C57BL/6 background. The APPPS1 mice were heterozygous and P2X7Rko mice were homozygous. Heterozygous APPPS1^+/Tg^xP2X7R^+/−^ mice were crossed with P2X7R^+/−^ mice to obtain the different groups of mice studied: WT (P2X7R^+/+^), P2X7Rko (P2X7R^−/−^), APPPS1 (APPPS1^+/Tg^), and APPPS1xP2X7Rko (APPPS1^+/Tg^xP2X7R^−/−^). Wild-type littermates served as control mice. Mice were housed under specific-pathogen-free conditions at the housing facilities of ICM.

For the different experiments, we selected animals at an age that displayed a significant difference between WT and APPPS1 animals, due to genotype.

Because sex can influence the development of AD pathology [29], we performed most experiments in male mice and confirmed the results in female mice, when relevant.

### Morris water maze (MWM)

Spatial memory was evaluated in 10-month-old WT (*n* = 11), P2X7Rko (*n* = 13), APPPS1 (*n* = 11), and APPPS1xP2X7Rko (*n* = 6) male mice with the MWM paradigm. The MWM included a 150-cm diameter pool filled with opaque water (21–22 °C). A 10-cm diameter platform was submerged 0.5 cm below the water surface in the center of one of the pool quadrants. The hidden platform remained at a constant position throughout the trials. Training consisted of one session (4 trials/session; start positions pseudo-randomly varied among the four cardinal points) every day for 7 consecutive days. Each trial ended when the animal reached the platform. The animal had a maximum of 60-s to reach the platform, after which it was manually guided to the platform. Once on the platform, the animal was given a 30-s rest before being returned to its cage. The inter-trial interval was ∼1 h. On the 8th training session, a probe test was performed. In this memory retention test, the platform was removed, and the mouse was allowed to navigate for 60-s.

Data were collected, analyzed, and stored with Any-Maze software (Stoelting Co., Wood Dale, IL, USA). We analyzed the corrected integrated path length (CIPL) parameter for each trial, which is the sum of the distances traveled by the mouse, from its start position to the platform, minus the distance the mouse would have traveled, swimming at its mean speed, along the shortest possible path between the start position and the platform [30]. CIPL is an unbiased measure for assessing learning over the seven training days. In addition, the evolution of navigation strategies (spatial vs. non-spatial) was assessed throughout the training sessions; the trajectories in each trial were manually classified by a trained experimenter in a blinded manner, according to previously defined criteria [31]. During the probe test, the percent of time spent in each quadrant was calculated to evaluate memory-related bias for the platform location.

### Tissue preparation

Mice were deeply anaesthetized and perfused transcardially with 50 mL PBS. The brains were removed from the skulls. One hemisphere was snap-frozen for biochemical analysis. The other was fixed by immersion in 4% paraformaldehyde, then cryoprotected. Hemispheres were sliced, either on a freezing microtome (40 µm serial sections) or on a cryostat (20 µm serial sections).

### RNAscope in situ hybridization (ISH)

RNAscope ISH (Advanced Cell Diagnostics) was performed with 20-µm sections of fixed frozen brain embedded in cryo-embedding medium. Tissues were pretreated by boiling in target retrieval solution for 5 min, then adding protease III for 30 min at 40 °C. After pretreatment, the slides were processed with the RNAscope multiplex fluorescent assay, according to manufacturer instructions. We used the probes designed by the manufacturer including Mm_P2X7R-C1, and positive and negative controls, Mm-PPIB and DapB (of the *Bacillus subtilis* strain), respectively. Immunofluorescence images were captured with an Apotome.2 system. Quantifications were performed with ImageJ software.

### Mouse tissue immunostaining

Aβ deposits were labeled by standard Congo red staining on 40 µm brain sections (30 min in an 80% ethanol solution saturated with Congo red and sodium chloride). Microscopic scans of whole sections (pixel size 0.25 μm²) were acquired with a NanoZoomer 2.0 RS slide scanner (Hamamatsu Photonics, Hamamatsu, Japan). Aβ loads were quantified with the spot detector plugin of the ICY software (http://icy.bioimageanalysis.org/), which automatically calculates the proportion of stained tissue (*p* = stained area/total area), thus providing unbiased stereological measurements. In addition, we measured the mean plaque size and quantified the density of plaques in each selected region of interest (ROI). All measurements were performed in the following ROIs: frontal cortex, hippocampus, sensorimotor cortex, and thalamus. For each ROI, we measured three sections per mouse and averaged the measurements.

For PSD95 immunostaining, free-floating 40 µm sections were incubated overnight at room temperature with rabbit polyclonal anti-PSD95 antibodies (#ab18258, Abcam, 1/1000 dilution). Then, the sections were incubated with biotinylated goat anti-rabbit IgG (H + L) antibodies for 2 h at room temperature (Vector Labs, 1/400 dilution). Sections were detected with the peroxidase–avidin–biotin technique (ABC Elite Kit, Vector Labs) with DAB as the chromogen. The time of revelation in DAB was the same for all tissues. After image digitization, the dorsal hippocampus and corpus callosum (reference area) were manually outlined on microphotographs. The optical density (OD) of each region was then assessed with MCID Elite image analysis software (Interfocus Technologies, Inc.). The hippocampus ODs were normalized to the corpus callosum to obtain the relative optical density (ROD).

Iba1 immunohistochemistry was performed as described above, and counterstaining was performed with Congo red dye. An anti-Iba1 rabbit polyclonal antibody (Wako, 1/3000 dilution) was used as a primary antibody. A general assessment of microglial cell density was first performed on scanner-digitized sections with ICY software. Images were segmented with the BestThreshold plugin, and the number of above-threshold pixels was counted in each ROI with a lab-customized script to provide a local “microglial load”.

The pattern of microglial accumulation around Aβ plaques was evaluated with published protocols [32]. Briefly, digitized sections of the frontal cortex of APPPS1 and APPPS1xP2X7Rko mice were imported into ImageJ. Ten plaques per mouse were outlined as circular ROIs, and the Concentric Circles plugin (https://imagej.nih.gov/ij/plugins/concentric-circles.html) was applied to create an overlay of five concentric circles. The innermost ring (ring 1) was centered on the Aβ core, and rings 2–5 were each enlarged by one additional plaque radius from the center (Fig. 3). Pixel intensities were measured and normalized between 0 (white) and 255 (black) within each ring to provide an estimate of regional cell–tissue occupancy (i.e., microglial densities).

Glial cell immunofluorescence was performed with 20-µm brain sections. Sections were incubated overnight at 4 °C with anti-Iba1 (Wako, 1/1000 dilution) and anti-GFAP (Dako, 1/1000 dilution) rabbit polyclonal antibodies. For T cell immunofluorescence, 20 µm brain sections were pretreated with 5 µg/mL proteinase K for 3 min at 37 °C and incubated overnight at 4 °C with anti-CD3 (Clone 17A2, R&D, 1/50 dilution), anti-CD4 (Clone YTS191.1, Bio-Rad, 1/100 dilution), and anti-CD8 (Clone KT15, Bio-Rad, 1/100 dilution) rat monoclonal antibodies. Sections were subsequently incubated with the appropriate Alexa Fluor secondary antibodies (Life Technologies, 1/1000 dilution, 2 h at room temperature). Images were acquired with an Apotome.2 system for fluorescence microscopy.

### Brain protein extraction

Snap-frozen brain hemispheres were homogenized in tissue protein extraction reagent (T-PER, Thermo Fisher Scientific) containing a mixture of protease and phosphatase inhibitors (Thermo Fisher Scientific). Homogenates were centrifuged at 100,000×*g* for 1 h at 4 °C. Brain protein concentrations were evaluated with a BCA assay (Thermo Fisher Scientific). The supernatants were evaluated for the quantification of Aβ peptides, cytokines, and chemokines (described below).

### ELISA quantification of cerebral Aβ peptides, cytokines, and chemokines

Cerebral Aβ peptides and cytokines were quantified with the MSD V-PLEX Human Aβ42 Kit, the V-PLEX Plus Aβ Peptide Panel 1 (6E10) Kit and the V-PLEX Plus Proinflammatory Panel 1 Mouse Kit according to the manufacturer’s instructions (Meso Scale Discovery). Signals were measured with a SECTOR Imager 2400 reader (Meso Scale Discovery). Cerebral chemokines were quantified with Chemokine 9-Plex Mouse ProcartaPlex™ Panel 1, according to the manufacturer’s instructions (eBioscience). Data acquisition was performed with a MAGPIX (Luminex). Each sample was measured in duplicate. Aβ peptides, secreted cytokines and chemokines were normalized to the total brain protein concentration, evaluated with a BCA assay.

### Western Blot analyses

Proteins in cell lysates were separated by SDS-PAGE and transferred to nitrocellulose membranes. Membranes blocked with 4% nonfat milk in Tris-buffered saline containing 0.2% Tween 20. Blots were immunostained with anti-APP (clone 22C11, Millipore) and anti-tubulin (clone B-5-1-2, Sigma-Aldrich) monoclonal antibodies at 4 °C overnight. Blots were probed with secondary antibodies conjugated to horseradish peroxidase (Invitrogen). Specific bands were visualized with enhanced chemiluminescence (Pierce). Quantification was performed with ImageJ software.

### Multi-electrode array (MEA)

Acute hippocampal slice preparations. Transverse 350 µm hippocampal slices were prepared from 16-month to 18-month-old male mice after pentobarbital anesthesia (140 mg/kg) and transcardial perfusion. Slices were cut with a vibrating blade microtome (VT1200S, Leica Biosystems) in a solution bubbled with 5% CO_2_ in 95% O_2_, cooled to 0–2 °C. This solution contained (in mM): 70 sucrose, 80 NaCl, 2.5 KCl, 1.25 NaH_2_PO_4_, 25 NaHCO_3_, 7 MgCl_2_, 0.5 CaCl_2_, 25 glucose, pH 7.3, osmolarity 315 mOsm. Slices were maintained at 20–25 °C in the following solution (in mM): 119 NaCl, 2.5 KCl, 26 NaHCO_3_, 1 NaH_2_PO_4_, 3 MgCl_2_, 2 CaCl_2_, 15 glucose; equilibrated with 5 % CO_2_ in 95 % O_2_, pH 7.3, 297 mOsm. For MEA recordings, hippocampal slices were transferred to an MED64 probe (Alpha MED Scientific Inc.) with a 150-µm inter-electrode distance and continuously perfused (3 mL/min) with ACSF containing (in mM) 119 NaCl, 5 KCl, 2.5 CaCl_2_, 1.3 MgCl_2_, 9.3 KH_2_PO_4_, 25 NaHCO_3_, and 5 glucose, saturated with 5% CO_2_ in 95% O_2_ at 32 °C.

MEA recordings. Field postsynaptic potentials (fPSPs) were recorded in the stratum pyramidale and stratum radiatum layers of the CA1 hippocampal region with an MEA system (MED64, Alpha MED Scientific). Schaffer collateral/commissural pathways were stimulated with biphasic current pulses (200 µs). First, we applied stimuli with increasing amplitudes from 10 to 100 μA to obtain Input–output (I/O) curves. Then, we applied stimulation intensity that elicited 40–50% of the maximum response to evoke fPSPs. Paired pulse facilitation (PPF) was measured with two stimulations applied at an inter-pulse interval varying from 20 to 200 ms. Percentage of facilitation was the fPSP slope elicited with the second pulse divided by the fPSP slope elicited with the first pulse. For the long-term potentiation (LTP) protocol, two 0.1 Hz stimulations were applied, with a 50-ms inter-stimulus interval. LTP was induced with a high-frequency stimulation (HFS) consisting of two trains at 100 Hz for 1 s, delivered 5 s apart, at 70–80% of the intensity that evoked the maximum fPSP. Before performing the HFS, a stable baseline was established for at least 15 min. The magnitude of LTP was quantified as the percentage change in the fPSP initial slope (10–40%), measured during the 45–60 min interval after HFS. Data were filtered at 1 kHz, digitized at 20 kHz and analyzed with Mobius software (Alpha Med Scientific).

### Isolation of brain-myeloid cells and flow cytometric analyses

To evaluate Aβ phagocytosis, 3 h before sacrifice, 12-month and 16-month-old female mice were injected intraperitoneally with methoxy-X04 (10 mg/kg in 50% DMSO/50% PBS-NaOH 0.1 N, Tocris Bioscience). The CNS-immune cells were isolated with Percoll separation, and cells were labeled as previously described [7]. Briefly, mice were perfused with PBS, and brain hemispheres were dissected and homogenized in a digestion cocktail containing the enzyme Liberase TL at 1.6 Wünsch unit/mL and DNAse1 at 0.5 mg/mL (Sigma-Aldrich) for 30 min at 37 °C in 5% CO_2_. After washing, the brain homogenate was centrifuged in a 30/37/70% Percoll gradient (GE Healthcare). Myeloid cells were recovered from the 37/70% Percoll interphase and washed. Cells were incubated with 1 µg of Fc-Block (anti-CD16/CD32 antibodies, eBioscience) to prevent antibodies from binding to Fc receptors. Cells were labeled with the following antibodies diluted in 0.5% PBS-BSA or 0.5% PBS-BSA/0.05% saponin (for intracellular staining): CD45 (clone 30F11, BD Biosciences), CD11b (clone M1/70, eBioscience), CD11c (clone N418, eBioscience), CD14 (clone Sa2-8, eBioscience), CD36 (clone 72-1, eBioscience), MHCII (clone M5/114.15.2), iNOS (clone 6, BD Biosciences), p40 (clone C17.8, eBioscience), and P2X7R (clone 1F11, BioLegend). To determine compensation settings, we used CompBeads (BD Biosciences), and corresponding antibodies. Isotype-matched control antibodies were used as negative controls. Cell-bound antibodies were detected with a FACSVerse analyzer (BD Biosciences), and the data were analyzed with FlowJo Software (FlowJo LLC).

### Assessment of phagocytosis

Microglial phagocytic activity was quantified in acute brain slices from 16-month-old male mice, as previously described [33]. Briefly, acute coronal brain slices were incubated in a suspension of FCS-coated fluorescent carboxylated microspheres (2 µm diameter, flash red, Bang Laboratories, Inc.) at a concentration of 1.1 × 10^7^ microspheres/mL for 60 min at 37 °C in HBSS and then washed and fixed with 4% paraformaldehyde. Different microsphere coating methods, including coating with bovine serum albumin, showed comparable results in this phagocytosis assay. Brain slices were stained with anti-Iba1 and thiazine red to visualize microglia and Aβ plaque cores, respectively. Samples were evaluated with a confocal laser scanning microscope (Nikon eclipse Ti-E) to determine the microglial phagocytic index, i.e. the quotient of internalized microspheres per total microglial cell number in each field of view. The microglial phagocytic index was determined in three corresponding ROIs per acute brain slice.

### Primary cell culture and stimulation of chemokine release

Primary cultures of microglia and astrocytes were prepared from the brain hemispheres of 1–3 days old. Cells were grown in culture medium containing DMEM, Glutamax, nonessential amino acids, sodium pyruvate, gentamycin, and 10% endotoxin-free fetal calf serum (Life Technologies). Cells were then plated in 10-cm culture dishes coated with poly-ornithine and incubated at 37 °C in a humid atmosphere with 5% CO_2_. The medium was changed weekly. After 2 weeks, microglia were recovered from the astrocyte layer by shaking (325 rpm) on an orbital shaker for 1 h at room temperature. Astrocytes were removed from the culture dishes with 0.05% trypsin-EDTA. Cells were washed and seeded at a density of 50,000 cells/well in 96-well plates. These conditions yielded nearly pure microglia and astrocyte populations. For cell stimulation, the medium was replaced with DMEM containing 0.5% BSA. Then, cells were stimulated, without or with LPS (*E. coli* 0111:B4, InvivoGen), ATP, Bz-ATP (Sigma-Aldrich) or Aβ peptides (Eurogentec) for 4 h at 37 °C and 5% CO_2_. LPS stimulation served as a positive control. Aβ peptides were dissolved in water at 1 mg/mL. For fibril formation, Aβ peptides were incubated for 7 days at 37 °C, then stored at −20 °C. Chemokines levels in the supernatant were assessed with mouse CCL3 DuoSet ELISAs according to the manufacturer’s instructions (R&D Systems).

### Statistics

All data are expressed as the mean ± SEM. Statistical analyses were performed with GraphPad Prism 6 (GraphPad Software), Statistica 10–13 (StatSoft, Inc.), and SigmaPlot (Systat Software, Inc.). Spatial navigation strategies in the MWM experiment were compared with Fisher’s exact test (2 × 2 contingency tables). MEA results were compared with two-way analysis of variance, followed by Tukey’s test (for IO curves and PPF) or with Kruskal–Wallis one-way ANOVA for ranks, followed by Dunn’s multiple comparison test for LTP. In other experiments, between-group differences were determined with one-way ANOVA, followed by Bonferroni’s post hoc test. Alternatively, the mean significant difference between two groups was determined with two-tailed unpaired Student’s *t*-test. Each figure legend specifies the statistical test used. Statistical significance was defined as: *P < 0.05, **P < 0.01, and ***P < 0.001. Outlier values detected with Grubbs’ test (GraphPad Software) were excluded from the analyses. Animals were allocated to experimental groups according to tgenotype. Data analyses were performed blinded to the genotype.

## Results

### Both AD patients and AD transgenic mouse model showed elevated brain P2X7R expression

**Fig. 1:**
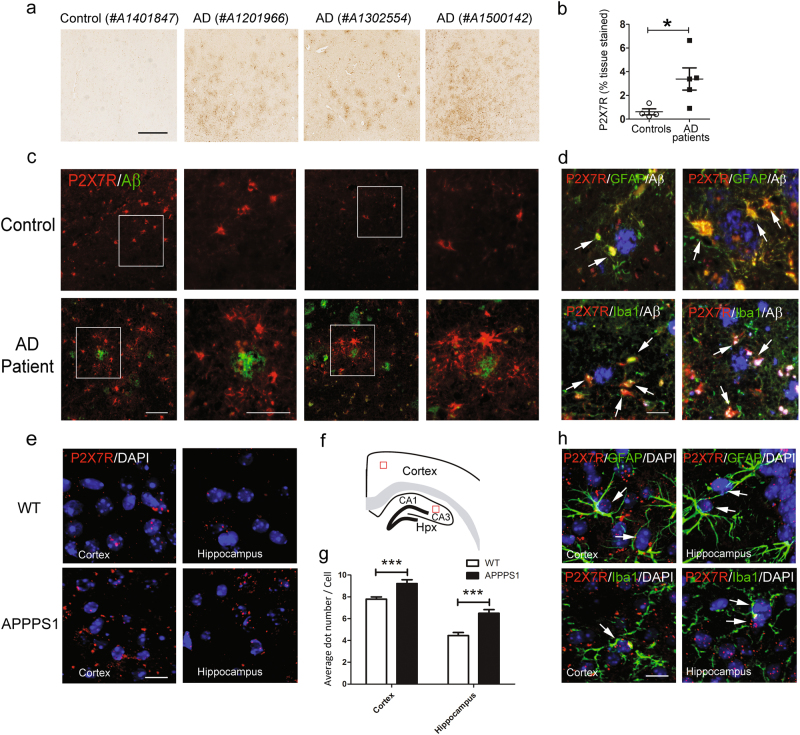
AD patients and mice express increased levels of P2X7R in the brain. **a** Image of brain slices from one non-demented human control and three AD patients stained with an anti-P2X7R antibody. Scale bar: 500 µm. **b** Quantification of P2X7R staining in the gray matter from non-demented human controls (*n* = 4) and AD patients (*n* = 5). Student’s *t*-test; *P < 0.05. **c** Images of brain slices from one non-demented control and one AD patient, double immunostained with anti-P2X7R (red) and anti-Aβ (green) antibodies. (right panels) Magnifications of the areas indicated with the white rectangle. Scale bar: 50 µm. **d** Images of brain slices from AD patients, double immunostained with anti-P2X7R (red) and either (upper panels) anti-GFAP (astrocyte marker, green) or (lower panels) anti-Iba1 (microglial marker, green). Scale bar: 25 µm. **e** Images of brain slices from WT (upper panels) and APPPS1 (lower panels) mice hybridized with RNAscope probes for detecting P2X7R mRNA (red). Nuclei were counterstained with DAPI (blue). Scale bar: 20 µm. **f** Schematic of mouse brain. Red-bordered squares in the cortex and hippocampus indicate areas in which dots were counted. **g** Quantification of the number of P2X7R mRNAs (dots) per cell in the cortex of WT (*n* = 50 cells) and APPPS1 (*n* = 54 cells) mice, and in the hippocampus of WT (*n* = 42 cells) and APPPS1 (*n* = 45 cells) mice. Student’s *t*-test; ***P < 0.001. **h** Images of brain slices from APPPS1 mice hybridized with RNAscope probes for detecting P2X7R mRNA (red) and immunostained with either (upper panels) anti-GFAP antibodies (green) or (lower panels) anti-Iba1 antibodies (green). Nuclei were counterstained with DAPI (blue). Scale bar: 20 µm

Previously, reverse transcriptase-polymerase chain reaction results showed that P2X7R expression was enhanced in AD patients [18]. Here, we aimed to confirm those results at the protein level. We performed immunohistochemical analyses of cortices from individuals without (control) or with AD. Analyses of P2X7R expression revealed a weak P2X7R signal in control subjects (0.61 ± 0.25% of tissues stained, *n* = 4) and stronger immunoreactivity in AD subjects (3.39 ± 0.94%, *n* = 5, Fig. 1a, b). Co-labeling with anti-Aβ and anti-P2X7R antibodies showed that P2X7R was overexpressed in the plaque cores and surrounding haloes (Fig. 1c). The cell types expressing P2X7R have not been clearly established in AD, thus we analyzed P2X7R expression further with double immunostaining for P2X7R and GFAP, an astrocyte marker, or Iba1, a microglia marker. We detected P2X7R expression in both astrocytes and microglia in the human brain (Fig. 1d).

To extend this work to the animal model we used, we performed RNAscope ISH to determine P2X7R mRNA expression levels in APPPS1 mice, instead of immunostaining, because the specificity of mouse P2X7R antibodies is controversial. We found that P2X7R mRNA expression was prominent in the cornu ammonis 3 (CA3) region of the hippocampus, as recently demonstrated by Metzger et al. [34]. (Supplementary Figure 1). Compared to wild-type mice, APPPS1 mice displayed higher P2X7R mRNA expression in the cortex (WT: 7.8 ± 0.2 dots/cell, *n* = 50; APPPS1: 9.2 ± 0.4 dots/cell, *n* = 54; *P* < 0.001) and hippocampus (WT: 4.5 ± 0.3 dots/cell, *n* = 42; APPPS1: 6.5 ± 0.3 dots/cell, *n* = 45; *P* < 0.0001; Fig. 1e–g). The RNAscope ISH assay was also used to detect P2X7R mRNA in astrocytes and microglia labeled with anti-GFAP and anti-Iba1 antibodies, respectively (Fig. 1f). We found that both astrocytes and microglia expressed P2X7R. These results were consistent with a previous study that analyzed P2X7R expression in conditional humanized mice [34]. Moreover, our results showed that glial P2X7R expression increased with Aβ pathology, both in patients with AD and in a mouse AD model.

### P2X7R deficiency reduces cognitive impairment and Aβ load in APPPS1 mice

In light of the observed increases in P2X7R expression in AD, we next examined whether decreased P2X7R expression might rescue AD pathologies; particularly the cognitive dysfunction that accompanies Aβ neuropathology. We used flow cytometry to confirm that macrophages and microglia from P2X7Rko mice did not express P2X7R. We also confirmed that LPS priming and ATP stimulation could not induce P2X7R-deficient microglia to release IL-1β, as previously described [28] (Supplementary Figure 2).

**Fig. 2:**
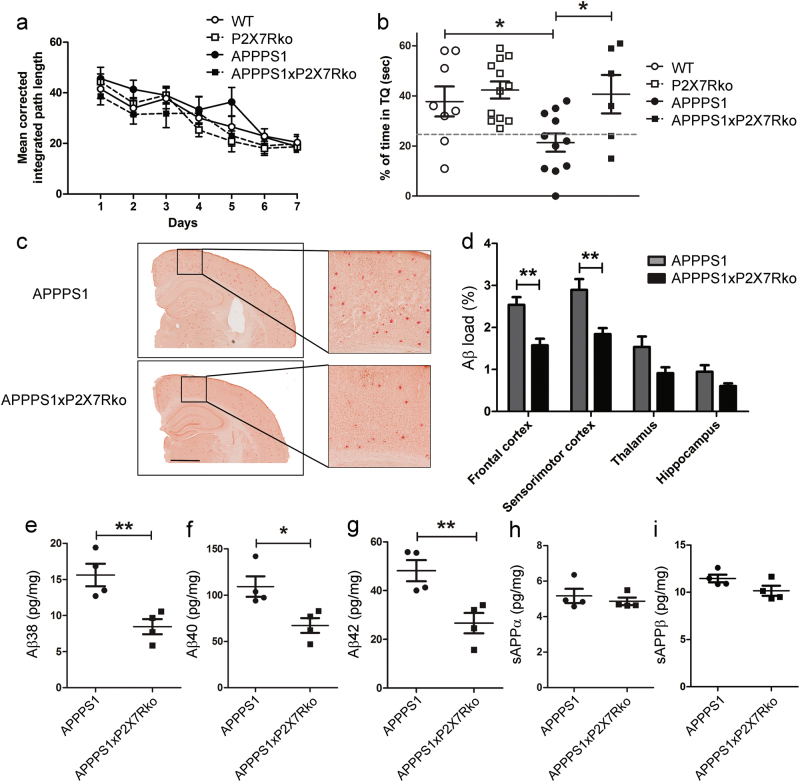
P2X7R deficiency improves memory impairments and decreases Aβ load in APPPS1 mice. **a** Analysis of learning performances in the Morris Water Maze, assessed with the mean corrected integrated path length (CIPL) taken to reach the hidden platform by 10-month-old male WT (*n* = 11), P2X7Rko (*n* = 13), APPPS1 (*n* = 11), and APPPS1xP2X7Rko (*n* = 6) mice. Two-way ANOVA with training session (*F* = 21.35) and genotype (*F* = 3.452) as the principal factors. **b** Percentage of time spent exploring the target quadrant (TQ) during the memory probe test. The target quadrant held the platform was located during training sessions: WT (*n* = 8), P2X7Rko (*n* = 12), APPPS1 (*n* = 11), and APPPS1xP2X7Rko (*n* = 6). One-way ANOVA followed by Bonferroni’s post hoc test (*F* = 4.78). *P < 0.05. **c** Representative images of brain slices from 10-month-old male APPPS1 and APPPS1xP2X7Rko mice stained with Congo red. Scale bar: 1 mm. **d** Quantification of Aβ load in different isocortical regions of 10-month-old male APPPS1 (*n* = 4) and APPPS1xP2X7Rko (*n* = 4) mice. Two-way ANOVA with brain region (*F* = 29.39) and genotype (*F* = 30.07) as the principal factors, followed by Bonferroni’s multiple comparison tests. **P < 0.01. **e**–**i** ELISA analyses of Aβ peptides, sAPPα and sAPPβ fragments in the brains of 10-month-old male APPPS1 (*n* = 4) and APPPS1xP2X7Rko (*n* = 4) mice. Student’s *t*-test. *P < 0.05 and **P < 0.01

We first determined spatial memory performance of 10-month-old WT, P2X7Rko, APPPS1 and APPPS1xP2X7Rko animals with the MWM test. All mice showed improvements in learning strategy throughout the training sessions, as shown by a decrease in the CIPL over time (*P* < 0.001; Fig. 2a, no effect of genotype). Although these findings suggested similar behavioral performances for all mice in the MWM, differences between the groups were apparent, when we analyzed the distribution of navigational strategies (Supplementary Figure 3). After completing all the training sessions, WT mice performed a significantly higher number of spatial trials versus non-spatial trials compared to APPPS1 mice (*P* < 0.01). In contrast, no differences were observed between WT and APPPS1xP2X7Rko mice (*P* > 0.592). These results indicated that the P2X7R deficiency rescued the impairment in navigational strategies in APPPS1 mice. We next performed a memory probe test. We found that APPPS1 mice explored all quadrants of the pool equally, which reflected a lack of specific memory of the goal location. Statistical analyses confirmed this memory impairment in APPPS1 mice compared to WT mice (*P* < 0.025, Fig. 2b). Importantly, APPPS1 mice also performed poorly compared to APPPS1xP2X7Rko mice (*P* < 0.025), and no differences were observed between WT and APPPS1xP2X7Rko mice (*P* > 0.766), emphasizing that P2X7R deficiency rescued the cognitive decline of APPPS1 mice (Fig. 2b).

We next examined whether the behavioral impact of P2X7R knockout correlated with changes in Aβ plaques. We used Congo red staining to analyze the distribution of Aβ plaques in 10-month-old male APPPS1 and APPPS1xP2X7Rko mice (Fig. 2c, d). We observed a higher load of Aβ plaques in the brains of APPPS1 mice compared to the brains of APPPS1xP2X7Rko mice (*P* < 0.01, Fig. 2d). Post hoc quantitative analyses confirmed this qualitative observation in the isocortical region (frontal cortex: APPPS1 2.54 ± 0.18% vs. APPPS1xP2X7Rko 1.58 ± 0.16%; sensorimotor cortex: APPPS1 2.90 ± 0.26% vs. APPPS1xP2X7Rko 1.84 ± 0.14%; between-genotype difference: *P* < 0.01). Complementary analyses indicated that the P2X7Rko-associated reduction in the Aβ plaque load was accompanied by a drastic reduction in the number of Aβ plaques (*P* < 0.0001), but the mean plaques size did not vary between APPPS1 and APPPS1xP2X7Rko mice (*P* > 0.281) (Supplementary Figure 4). Parallel experiments were performed in female mice (8-month-old), and similar results were obtained; the overall Aβ load was decreased in APPPS1xP2X7Rko mice compared to APPPS1 mice (Supplementary Figure 5a, b).

Because soluble Aβ species cause severe synaptotoxicity in APPPS1 mice [35], we next performed ELISAs to analyze the soluble Aβ peptide levels in whole brain extracts from APPPS1xP2X7Rko vs. APPPS1 mice. We found significant lower Aβ peptide concentrations in 10-month-old APPPS1xP2X7Rko mice (Aβ38: 8.4 ± 1.0 pg/mg, Aβ40: 67.4 ± 8.0 pg/mL, Aβ42: 26.6 ± 4.2 pg/mL, *n* = 4) vs. APPPS1 mice (Aβ38: 15.6 ± 1.6 pg/mg, Aβ40: 109.4 ± 11.1 pg/mL, Aβ42: 48.2 ± 4.3 pg/mL, *n* = 4, Fig. 2e–g). In a previous in vitro study, we demonstrated that P2X7R activation stimulated the beneficial non-amyloidogenic processing of APP into sAPPα [23]. Thus, we also determined the levels of sAPPα and sAPPβ in whole brains of APPPS1xP2X7Rko vs. APPPS1 mice. We found similar levels of sAPPα and sAPPβ fragments between these groups. These results indicated that P2X7R deficiency did not modulate APP processing in APPPS1 mice (Fig. 2h, i). In parallel experiments, and like their male counterparts, we also observed a decrease in soluble Aβ40 and Aβ42 levels in the brains of 8-month-old female APPPS1xP2X7Rko compared to APPPS1 mice, and similar levels of sAPP fragments between genotypes (Supplementary Figure 5c–g). To investigate the possibility that P2X7R-deficiency might modulate the expression of APP, which could lead to a reduction of Aβ production, we compared total APP levels in the brains of APPPS1 and APPPS1xP2X7Rko mice. We showed that APP levels were not significantly different between APPPS1xP2X7Rko and APPPS1 mice (Supplementary Figure 6).

In summary, P2X7R-deficiency rescued memory deficits and reduced the Aβ load in APPPS1 mice.

### P2X7R-deficiency rescues synaptic dysfunction in APPPS1 mice

Next, we asked whether the observed phenotypic changes in Aβ plaques and behavior might be associated with differences in synaptic function.

**Fig. 3:**
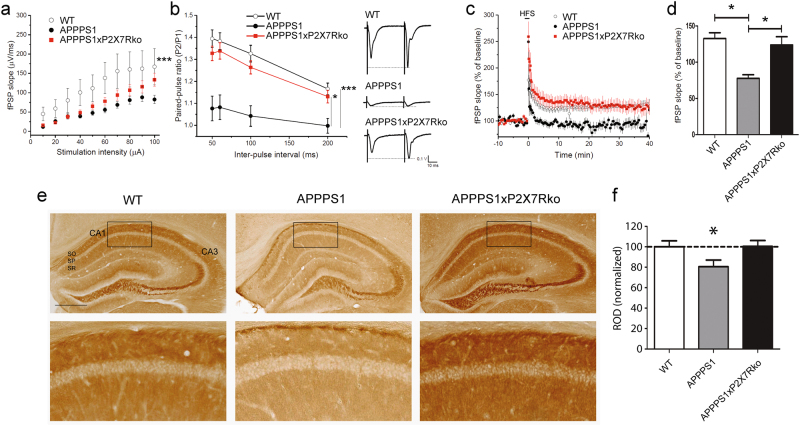
Deficits in short-term synaptic plasticity and LTP in APPPS1 mice are rescued in APPPS1xP2X7Rko mice. Results from the multi-electrode array (MEA). Field postsynaptic potentials (fPSPs) were evoked by stimulating Schaffer collateral fibers and recorded in the CA1 stratum radiatum in slices obtained from 18-month-old WT (*n* = 5), APPPS1 (*n* = 5), and APPPS1xP2X7Rko (*n* = 5) mice. **a** Input-output curve of fPSP slope versus stimulus intensity. Two-way ANOVA with stimulation intensity (*F* = 6.86) and genotype (*F* = 20.93) as the principal factors, followed by Tukey’s post hoc test. fPSP slope: ***P ≤ 0.001 WT vs. APPPS1 and *P* = 0.210 APPPS1xP2X7Rko vs. APPPS1. **b** Paired-pulse ratios (PPRs) measured at different inter-stimulus intervals (50, 60, 100, and 200 ms) were significantly reduced in APPPS1 mice. Two-way ANOVA with inter-pulse interval (*F* = 8.04) and genotype (*F* = 61.23) as the principal factors, followed by Tukey’s post hoc test. ***P ≤ 0.001 WT vs. APPPS1. Moreover, PPR was significantly different between APPPS1xP2X7Rko and APPPS1 mice. *P < 0.05. Right: representative traces of fPSPs evoked by two stimulations with a 50 ms interval in WT, APPPS1 and APPPS1xP2X7Rko mice (stimulus artifact removed). **c**, **d** Long-term potentiation (LTP) was induced by two bursts of high-frequency stimuli (HFS: 100 pulses at 100 Hz), delivered with an interval of 5 s. LTP was significantly reduced in APPPS1 mice compared to WT mice, but rescued in APPPS1xP2X7Rko mice; Kruskal–Wallis one-way ANOVA by ranks followed by Dunn’s multiple comparison test (*F* = 8.38) (*n* = 5 per group, average of 5 min at 40 min). *P *<* 0.05. **e** Immunohistochemistry images of brain slice stained with anti-PSD95 antibodies show post-synaptic integrity in the hippocampus of 18-month-old mice. CA cornu ammonis SO stratum oriens, SP stratum pyramidale, SR stratum radiatum. (Lower panels) Magnifications of areas indicated with black rectangles. Scale bar: 500 µm. **f** Quantification of PSD95 staining in the hippocampus of WT (*n* = 7), APPPS1 (*n* = 5), and APPPS1xP2X7Rko (*n* = 6) mice. Means represent least three sections per mouse. Student’s *t*-test. *P *<* 0.05

To address this question, we used a MEA technique to assess synaptic transmission in the stratum radiatum of the CA1 hippocampus of 18-month-old WT, APPPS1 and APPPS1xP2X7Rko mice. We first observed an alteration in basal synaptic transmission between CA3 and CA1 neurons in hippocampal slices from APPPS1 vs. WT mice (synaptic strength was reduced by 85%, *P* < 0.018 at the stimulation intensity of 100 μA, Fig. 3a). The I/O curve was slightly but not significantly increased in APPPS1xP2X7Rko vs. APPPS1 mice (difference of the mean: 51%, *P* = 0.287 at 100 μA). These results implied that differences in basal synaptic transmission in the CA1 region were not rescued by the genetic knockdown of P2X7R.

Next, we investigated paired-pulse facilitation (PPF), a form of short-term synaptic plasticity that occurs in the CA1 region. Interestingly, PPF was not readily observed in APPPS1 mice (PPF ratio = 1.07 with an inter-stimulus interval of 50 ms), thus PPF was strongly decreased relative to WT (PPF ratio = 1.39, inter-stimulus interval of 50 ms) and APPPS1xP2X7Rko (PPF ratio = 1.32, inter-stimulus interval of 50 ms, *P* ≤ 0.001; Fig. 3b) mice. Interestingly, the PPF ratio was similar between APPPS1xP2X7Rko and WT mice at all tested inter-stimulus intervals. These results indicated that P2X7R deficiency protected against alterations of the presynaptic mechanism of PPF observed in APPPS1 mice.

Finally, we investigated long-term synaptic plasticity by assessing LTP induced by HFS in the CA1 region. Hippocampal LTP was considerably reduced in APPPS1 vs. WT mice (Fig. 3c), and LTP was significantly recovered in APPPS1xP2X7Rko mice (Fig. 3c). More precisely, at 40 min post-HFS, the magnitude of LTP was 77.7 ± 5.0% in APPPS1 mice compared to 132.7 ± 8.0% and 123.8 ± 11.4% in WT and APPPS1xP2X7Rko mice, respectively (all values relative to baseline; Fig. 3d).

To complement this functional data, we evaluated synaptic integrity by quantifying PSD95 immunostaining. Compared to WT mice (100.0 ± 5.8 %, *n* = 7), APPPS1 mice displayed decreased PSD95 immunoreactivity (80.6 ± 6.6%, *n* *=* 5, *P* < 0.05), and this decrease was prevented by P2X7R deficiency (100.4 ± 5.8 %, *n* = 6, Fig. 3e, f).

Taken together, these findings showed that P2X7R deficiency rescued the alterations in synaptic integrity and the LTP deficits observed in APPPS1 mice.

### P2X7R deficiency does not influence microglial activation or cytokine production

Based on our observations that P2X7R knockout decreased AD-related pathology in a mouse model and given that P2X7R activation-induced microglial activation and cytokine release [10, 28, 36], we next asked whether knocking out this receptor would also decrease the pro-inflammatory effects previously reported in mouse models of AD [37]. To that end, we first investigated whether P2X7R played a role in the altered microglial properties characteristic of APPPS1 mice [7, 33].

**Fig. 4:**
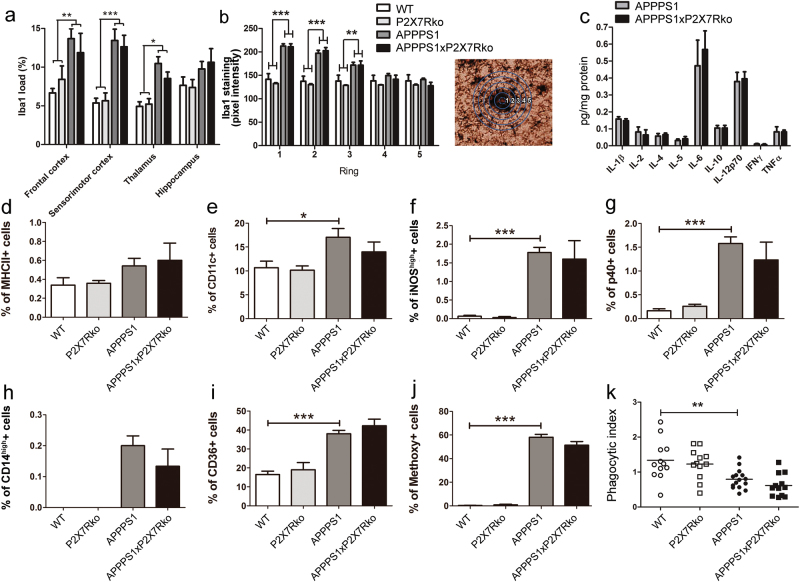
P2X7R deficiency does not affect microglia recruitment, phenotype, phagocytic properties, or cytokine release. **a** Analysis of Iba1 load in 10-month-old WT (*n* = 8), P2X7Rko (*n* = 8), APPPS1 (*n* = 5), and APPPS1xP2X7Rko (*n* = 4) male mice. **b** Periplaque microglial recruitment in 10-month-old WT (*n* = 3), P2X7Rko (*n* = 3), APPPS1 (*n* = 5), and APPPS1xP2X7Rko (*n* = 4) male mice. (Right panel) An overlay of five concentric circles centered on the Aβ core was applied to determine Iba1 load as a function of the distance from the center of the plaque. At least three sections per mouse were used for Iba1 load analysis and 10 plaques per mouse were used for microglial recruitment analysis. Two-way ANOVA with brain region (*F* = 3.98) and genotype (*F* = 23.87) (**a**) or ring (*F* = 22.23) and genotype (*F* = 48.19) (**b**) as the principal factors, followed by Bonferroni’s multiple comparison tests. *P < 0.05, **P < 0.01, ***P < 0.001. **c** Multiplex ELISA results show cytokine protein expression in the brains of 10-month-old APPPS1 (*n* = 4) and APPPS1xP2X7Rko (*n* = 4) male mice. Student’s *t*-test. Percentage of CD11b^+^CD45^med^ microglial cells expressing MHCII (**d**), CD11c (**e**), iNOS (**f**), p40 (**g**), CD14 (**h**), CD36 (**i**) and methoxy-X04 (**j**) from 12-month-old WT (*n* = 8), P2X7Rko (*n* = 7), APPPS1 (*n* = 5) and APPPS1xP2X7Rko (*n* = 6) mice. **k** Phagocytic activity of microglial cells (Iba1+) was investigated from 15-month-old WT (*n* = 4), P2X7Rko (*n* = 4), APPPS1 (*n* = 5), and APPPS1xP2X7Rko (*n* = 4) mice. *n* = 3 slices per mouse. One-way ANOVA followed by Tukey’s multiple comparison test (*F* = 8.79). *P < 0.05, **P < 0.01, ***P < 0.001

First, we characterized microglial distribution and periplaque accumulation by immunohistochemistry for Iba1. Transgenic mice (both APPPS1 and APPPS1xP2X7Rko mice) presented an increased Iba1 load in the cortex and the thalamus compared to WT mice (*P* < 0.001; Fig. 4a). The Iba1 load was not significantly different in APPPS1xP2X7Rko (frontal cortex: 11.9 ± 2.5%, *n* = 4) and APPPS1 mice (frontal cortex: 13.7 ± 1.3%, *n* = 5, Fig. 4a). Furthermore, analysis of periplaque microglial recruitment was consistent with the quantification of Iba1 loads. In APPPS1 mice, the microglia clustered mostly around the core of the Aβ plaque (ring 1) and at periplaque positions (rings 2 and 3). In APPPS1xP2X7Rko mice, the microglia recruitment was not significantly different from that in APPPS1 mice (ring 1, APPPS1: 212.3 ± 4.8, *n* = 5; APPPS1xP2X7Rko: 211.0 ± 6.3, *n* = 4; Fig. 4b). These data indicated that P2X7R deficiency did not affect microglia periplaque accumulation.

Microglia contribute to AD neurodegenerative processes via cytokine release. Previous studies have shown that P2X7R regulated the production of cytokines, including IL-1β, in vivo, after an intraperitoneal injection of LPS [28] or an intrahippocampal injection of Aβ peptides [10]. Thus, we investigated whether the cerebral cytokine levels of APPPS1xP2X7Rko mice differed from those of APPPS1 mice. We found no significant differences in the expression of cytokines studied in 10-month-old male APPPS1xP2X7Rko and APPPS1 mice (Fig. 4c). Similar results were obtained with 8-month-old females; P2X7R deficiency did not rescue microglia periplaque accumulation or affect IL-1β production in APPPS1 mice in the absence of external priming (Supplementary Figure 2h–j). Comparable levels of IL-1β were also observed between APPPS1xP2X7Rko and APPPS1 mice in 12-month-old females and 15-month-old males (Supplementary Figure 7a, b). However, a P2X7R upsurge can arise from astroglia; therefore, we quantified astrogliosis in the brain of AD transgenic animals. We found that the GFAP signal density was not significantly different in the brains of APPPS1xP2X7Rko and APPPS1 mice (Supplementary Figure 7c, d).

We previously showed that microglial cells adopted a chronically activated phenotype with AD progression [7]. To determine the impact of P2X7R deficiency on the phenotype of microglia in APPPS1 mice, we analyzed the expression of MHCII and CD11c, two surface proteins involved in antigen presentation, which are known to be upregulated in activated myeloid cells. We also analyzed the percentages of iNOS^high^ microglia, which represent an extreme pro-inflammatory subpopulation, and cells that express the p40 subunit of IL-12 and IL-23 cytokines. We found that MHCII expression was not significantly different between genotypes (Fig. 4d), the percentages of CD11c^+^, iNOS^high^, and p40^+^ microglia were increased in APPPS1 mice (CD11c: 17.1 ± 1.8%, iNOS^high^: 1.78 ± 0.14%, *n* = 5) vs. WT mice (CD11c: 10.7 ± 1.4%, iNOS^high^: 0.06 ± 0.03%, *n* = 8) (Fig. 4e–g). The distribution of the different microglial subpopulations was not significantly different in APPPS1xP2X7Rko (CD11c: 14.0 ± 2.1%, iNOS^high^: 1.60 ± 0.50%, *n* = 6) vs. APPPS1 mice (Fig. 4e–g). Therefore, these results suggested that P2X7R did not modulate the activation state of microglia in APPPS1 mice.

To evaluate the impact of P2X7R deficiency on microglial phagocytosis properties, we next analyzed the expression of two receptors involved in Aβ phagocytosis. Consistent with our previous observations [7], the percentages of microglia expressing CD14 and CD36 were considerably higher in APPPS1 (CD14: 0.20 ± 0.03%, CD36: 38.1 ± 1.7%, *n* = 5) vs. WT mice (CD14: 0%, CD36: 16.5 ± 1.8%, *n* = 8) (Fig. 4h, i). However, the proportion of microglia expressing CD14 and CD36 were similar in the brains of APPPS1xP2X7Rko (CD14: 0.14 ± 0.06%, CD36: 42.3 ± 3.4%, *n* = 6) vs. APPPS1 mice (Fig. 4h, i).

Furthermore, we evaluated the effect of P2X7R deficiency on the phagocytosis of Aβ peptides in vivo. We labeled Aβ-phagocytic microglia, by injecting mice with methoxy-X04, a fluorescent derivative of Congo Red known to cross the blood brain barrier and to bind Aβ with high affinity [20]. The proportion of methoxy-X04-labeled microglia in APPPS1 mice (58.1 ± 2.4%, *n* = 5) was not significantly different from that in APPPS1xP2X7Rko mice (51.3 ± 3.2%, *n* = 6) (Fig. 4j). Moreover, monocyte-derived macrophages from APPPS1xP2X7Rko mice also presented the same phenotype as APPPS1 mice (Supplementary Figure 8a). In addition, we observed no changes in the phenotypes of monocyte-derived macrophages or microglia in older mice (Supplementary Figure 8b, c).

Finally, microglial phagocytic capacity was analyzed in acute brain slices treated with fluorescent polystyrene microparticles (Supplementary Figure 9). The phagocytic index of cortical microglia was reduced in acute slices from APPPS1 mice (0.79 ± 0.07, *n* = 15 slices from five mice) vs. WT mice (1.34 ± 0.16, *n* *=* 12 slices from four mice), confirming previous reports [33]. However, statistical analyses did not reveal any significant differences in the phagocytic index between APPPS1 and APPPS1xP2X7Rko mice (0.61 ± 0.09, *n* = 12 slices from 4 mice) (Fig. 4k). Thus, P2X7R deficiency did not affect microglial Aβ uptake in APPPS1 mice.

In summary, P2X7R deficiency did not modify the microglial phenotype, cytokine secretion profile, or phagocytic activity of APPPS1 mice.

### P2X7R modulates cerebral chemokine production and contributes to CD8^+^ T cell recruitment in the brains of APPPS1 mice

**Fig. 5:**
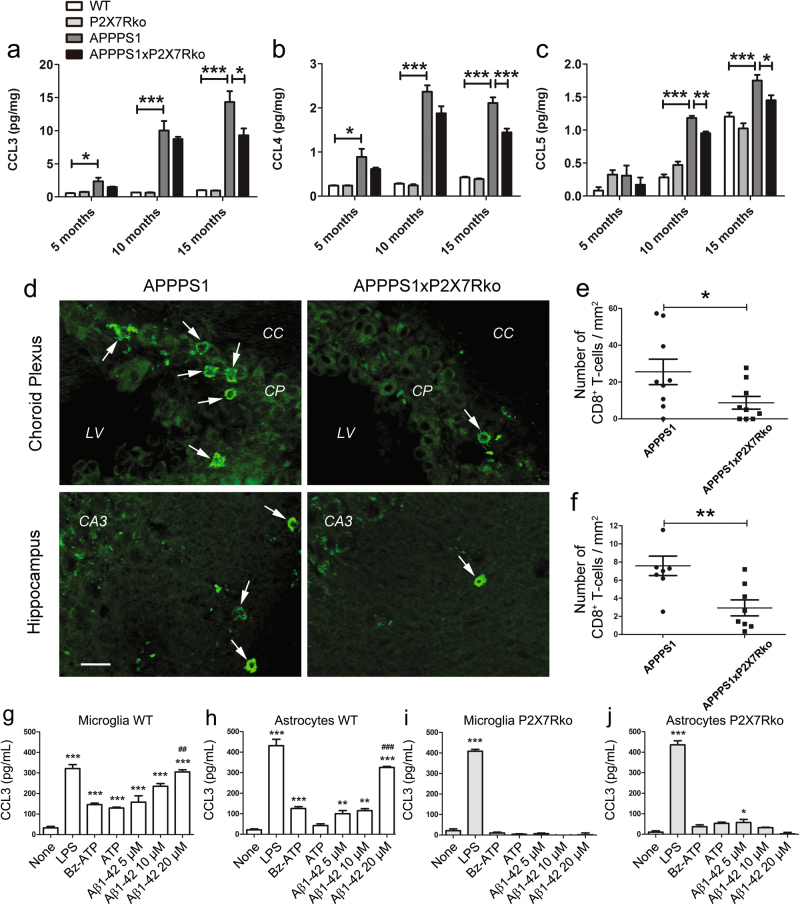
P2X7R deficiency decreases CCL3, CCL4, and CCL5 production and CD8^+^ T cell recruitment induced by Aβ peptides. Multiplex ELISA results show CCL3 (**a**), CCL4 (**b**), and CCL5 (**c**) protein expression in the brains of 5-month, 10-month, and 15-month-old WT (*n* = 4–8), P2X7Rko (*n* = 3–6), APPPS1 (*n* = 4–11), and APPPS1xP2X7Rko (*n* = 4–10) mice. One-way ANOVA followed by Tukey’s multiple comparison test. *P < 0.05, **P < 0.01, ***P < 0.001. **d** Labeled CD8^+^ T cells in brain sections from 15-month-old APPPS1 and APPPS1xP2X7Rko mice. CC corpus callosum, CP choroid plexus, LV left ventricle, CA3 cornu ammonis 3. Scale bar: 20 µm. The number of CD8^+^ T cells was determined in the choroid plexus (**e**) and hippocampus (**f**) of APPPS1 (*n* = 9) and APPPS1xP2X7Rko mice (*n* = 9). Unpaired Student’s *t*-test. *P < 0.05, **P < 0.01. ELISA results show CCL3 release in primary cultures of microglia (**g**–**i**) and astrocytes (**h**–**j**) from WT (**g**, **h**) or P2X7Rko (**i**, **j**) animals. Cells were stimulated for 4 h with LPS (100 ng/mL), or with Bz-ATP (300 µM), ATP (1 mM), or Aβ1–42 peptides (5, 10 or 20 µM). One-way ANOVA followed by Dunnett’s multiple comparison test ((**g**) *F* = 42.33; (**h**) *F* = 109.0; (**i**) *F* = 570.3; (**j**) *F* = 196.0). *P < 0.05, **P < 0.01, ***P < 0.001, ^##^*P* > 0.01, ^###^*P* < 0.001. Data are representative of at least three independent experiments

Chemotactic cytokines (chemokines) are overexpressed in response to Aβ peptides in vitro and in mouse models of AD. In AD, chemokines contribute to inflammatory processes and recruitment of immune cells [38] and consequent neurodegenerative processes [39]. Therefore, we hypothesized that the improvement in synaptic integrity and cognitive function observed in the P2X7Rko AD mouse model could be due to reduced chemokine secretion. To test this hypothesis, we performed multiplex ELISAs to compare cerebral levels of a panel of chemokines in APPPS1 and APPPS1xP2X7Rko male mice at 5, 10, and 15 months of age. As expected, we found elevated levels of CCL3, CCL4, and CCL5, particularly in 10-month and 15-month-old APPPS1 mice (15-month-old, CCL3: 14.3 ± 1.6 pg/mg, CCL4: 2.1 ± 0.1 pg/mg and CCL5: 1.8 ± 0.1 pg/mg, *n* = 11; *P* < 0.001) vs. WT mice (15-month-old, CCL3: 1.0 ± 0.1 pg/mg, CCL4: 0.4 ± 0.1 pg/mg and CCL5: 1.2 ± 0.1 pg/mg, *n* = 8; Fig. 5a–c). Interestingly, the levels of CCL3, CCL4, and CCL5 were lower in APPPS1xP2X7Rko (CCL3: 9.3 ± 1.1 pg/mg, *P* < 0.05; CCL4: 1.5 ± 0.1 pg/mg; *P* < 0.001, CCL5: 1.5 ± 0.1 pg/mg; *P* < 0.05; *n* = 10) vs. APPPS1 mice at 15 months of age (Fig. 5a–c). The levels of CCL2, CCL7, CCL11, CXCL1, CXCL2, and CXCL10, were significantly elevated in the brains of APPPS1 vs. WT mice but were not significantly different between APPPS1 and APPPS1xP2X7Rko mice (Supplementary Figure 10).

We next investigated whether the P2X7R-deficiency-induced changes in cerebral chemokine levels impact T cell recruitment in APPPS1 mice. First, we showed that CD3^+^ T cells were present in APPPS1 mouse brains (Supplementary Figure 11a,b). Then, we analyzed the number of CD4^+^ and CD8^+^ T cells in the choroid plexus (CP) and hippocampus of 15-month-old APPPS1xP2X7Rko and APPPS1 mice with immunohistochemistry. We observed numerous CD8^+^ T cells in the CP, which serves as a gate for controlling immune cell trafficking into the CNS, but few CD8^+^ T cells in the hippocampus of both APPPS1 and APPPS1xP2X7Rko mice (Fig. 5d–f). Nevertheless, compared to APPPS1 mice, APPPS1xP2X7Rko mice exhibited significantly lower CD8^+^ T cells densities in the CP and hippocampus (CP, APPPS1: 25.5 ± 6.9 cells/mm^2^, *n* = 9; APPPS1xP2X7Rko: 8.7 ± 3.5 cells/mm^2^, *n* = 9; *P* < 0.05; hippocampus, APPPS1: 7.6 ± 1.1 cells/mm^2^, *n* = 9; APPPS1xP2X7Rko: 2.9 ± 0.9 cells/mm^2^, *n* = 9; *P* < 0.01), indicating that P2X7R deficiency reduced CD8^+^ T cell recruitment into these neural regions in APPPS1 mice. Similarly, few CD4^+^ T cells were detected in the CP of APPPS1 mice (18.4 ± 4.5 cells/mm^2^, *n* = 6), and a lower number was observed in APPPS1xP2X7Rko mice (5.5 ± 2.9 cells/mm^2^, *n* = 5; *P* < 0.05); but, CD4^+^ T cells were barely detectable in the hippocampus (Supplementary Figure 11c–e). We also assessed the proportion of microglia and monocyte-derived macrophages in APPPS1 vs. APPPS1xP2X7Rko mice with flow cytometry. We found that P2X7R deficiency did not affect microglia/macrophage recruitment at 12 and 16 months of age (Supplementary Figure 11f-h).

Because CCL3 was one of the most highly upregulated (∼15-fold) cytokines in the brains of APPPS1 mice compared to WT mice, we performed a more extensive study of CCL3. The upregulation of CCL3 was confirmed in 12-month-old female mice (APPPS1: 42.7 ± 3.7 pg/mg, *n* = 6; APPPS1xP2X7Rko: 30.0 ± 1.8 pg/mg, *n* = 5; *P* < 0.05) and in 18-month-old male mice (APPPS1: 20.1 ± 0.7 pg/mg, *n* = 3; APPPS1xP2X7Rko: 16.8 ± 0.9 pg/mg, *n* = 4; *P* < 0.05) (Supplementary Figure 11i,j).

We previously demonstrated that CCL3 was expressed in astrocytes, microglia, and to a lesser extent, neurons of APPPS1 mice [40]. Therefore, to demonstrate the specific role of P2X7R in CCL3 production, microglial and astrocytic cell cultures from WT and P2X7Rko mice were stimulated with P2X7R agonist, ATP, and the more potent agonist, benzoyl-ATP (Bz-ATP), for 4 h and analyzed for CCL3 release by ELISA. Stimulation with P2X7R agonists led to an increase in CCL3 concentrations in the supernatant of microglia (Bz-ATP: 146.1 ± 7.2 pg/mL; ATP: 129.4 ± 4.7 pg/mL; *P* < 0.001; Fig. 5g) and astrocytes (Bz-ATP: 125.8 ± 9.0 pg/mL; ATP: 42.9 ± 8.0 pg/mL; *P* < 0.001; Fig. 5h) from WT mice vs. unstimulated controls (microglia: 32.7 ± 7.1 pg/mL; astrocytes: 21.9 ± 4.7 pg/mL). Microglia and astrocytes from P2X7Rko mice, however, failed to produce CCL3 in response to Bz-ATP (microglia: 10.1 ± 4.2 pg/mL; astrocytes: 37.1 ± 8.9 pg/mL) and ATP (microglia: 4.1 ± 2.3 pg/mL; astrocytes: 53.2 ± 5.8 pg/mL) (Fig. 5i, j). Moreover, we found that fibrillar Aβ1-42 stimulation led to a robust, dose-dependent release of CCL3 in supernatants from WT microglia (Aβ1-42, 5 µM: 157.7 ± 31.4 pg/mL; 10 µM: 235.2 ± 13.4 pg/mL; 20 µM: 305.1 ± 10.4 pg/mL; *P* < 0.01; Fig. 5g) and WT astrocytes (Aβ1-42, 5 µM: 100.1 ± 15.6 pg/mL; 10 µM: 115.0 ± 9.7 pg/mL; 20 µM: 325.7 ± 5.1 pg/mL; *P* < 0.001; Fig. 5h). Interestingly, the production of CCL3 was not elevated in the supernatants from P2X7Rko microglia or astrocytes stimulated with fibrillar Aβ1-42 (Aβ1-42, 20 µM: 2.2 ± 7.0 pg/mL and 1.1 ± 9.5 pg/mL, respectively; Fig. 5i, j).

Taken together, these results indicated that P2X7R was involved in Aβ peptide-induced chemokine production and CD8^+^ T cell recruitment in the brain parenchyma.

## Discussion

In this study, we investigated whether the lack of P2X7R promoted or prevented pathology in mouse model of AD. Furthermore, we analyzed the mechanism underlying the effects. We showed that P2X7R deficiency rescued memory deficits, restored hippocampal synaptic integrity and plasticity and reduced Aβ pathology in APPPS1 mice. However, these effects were not mediated by modulating the sAPPα pathway, IL-1β processing, microglial activation, or phagocytosis. Interestingly, our data highlighted the particular involvement of P2X7R in AD development by reducing chemokine release, and thereby decreasing T-cell recruitment. Based on these findings, we propose a new model of mechanistic pathways that explain the deleterious effects of inflammation in AD. In this model, chronically elevated levels of Aβ peptide induce ATP release from microglia and astrocytes [10, 11], subsequently, this ATP release is amplified by astrocytes via purinergic receptors [41]. In turn, P2X7R sense ATP and, in response, trigger parts of the AD neurodegenerative processes via chemokine production, leading to altered neuronal functions, damaged neurites, and pathogenic T cells recruitment (Supplementary Figure 12).

Based on the results from our previous in vitro study [23], we expected the global in vivo blockade of P2X7R signaling to decrease sAPPα release and concomitantly elevate Aβ production, resulting in worsening of AD pathology. In contrast, we observed that P2X7R deficiency in APPPS1 mice led to decreased Aβ peptide levels and reduced Aβ load, with no effect on sAPPα levels. Our data were consistent with findings in another Aβ model (J20), where treatment with the P2X7R antagonist, Brilliant Blue-G (BBG), caused a decrease in Aβ plaques [42]. Our results suggested that the lack of P2X7R did not modify the phagocytic properties of microglia; thus, the observed reduction in Aβ levels in APPPS1xP2X7Rko mice compared to APPPS1 mice were most likely due to effect on APP processing. Indeed, Leon-Otegui et al. showed, in vitro, that P2X7R stimulation for 4 h led to a reduction in α-secretase activity, which was the opposite of the effect observed with brief stimulation (<30 min) [23, 43]. Thus, we hypothesize that short P2X7R stimulation may induce beneficial sAPPα release, while sustained P2X7R activation in the Aβ mouse model may shift toward more toxic Aβ production. Another hypothesis to explain the reduction in Aβ peptides found in APPPS1xP2X7Rko mice, could be that P2X7R down-modulated the levels of proteolytic enzymes involved in Aβ peptide clearance, such as insulin-degrading enzyme. This biochemical pathway was claimed to be involved in the reduction of Aβ levels observed, when APP/PS1 mice were backcrossed to NLRP3 or caspase1-deficient mice [20]. We performed a western-blot analysis and found that the levels of insulin-degrading enzyme were similar in brain lysates of APPPS1 and APPPS1xP2X7Rko mice (data not shown). However, this experiment could not rule out the potential involvement of other proteolytic enzymes.

Furthermore, although P2X7R is a regulator of NLRP3 activation, we did not observe significant changes in IL-1β release in APPPS1xP2X7Rko mice. A previous study reported that P2X7R played a role in Aβ-mediated IL-1β secretion. After knocking-out P2X7R, they found reduced IL-1β levels in Aβ-treated cultured microglia and in Aβ-injected mice [10]. First, in vitro experiments showing IL-1β release were performed after LPS pre-stimulation. However, we observed that IL-1β release was not detected in response to ATP or Aβ peptide treatment in the absence of LPS pre-stimulation (data not shown). These data suggested that P2X7R did not induce IL-1β release without external priming; for example, with LPS [28] or an acute hippocampal injection [10]. The discrepancies between these studies may be due to the different murine Aβ models used. In one study, acute Aβ stimulation could have induced brief ATP release, which then led to P2X7R-dependent microglial activation; thus, this activation could be reduced by inhibiting P2X7R [10, 44]. However, in a chronic Aβ mouse model, sustained ATP release may induce the engagement of different purinergic receptors; thus, P2X7R deficiency might not alter microglial activation (Fig. 4) [45]. Indeed, depending on the cell type, other purinergic receptors can drive inflammasome activation. Notably, P2X4R is also involved in IL-1β release from ATP-stimulated primate microglia [46]. Furthermore, in the extracellular space, ATP is converted to adenosine by ectonucleotidases, which can also promote IL-1β production in macrophages via NLRP3 activation [12, 13]. Thus, the lack of P2X7R in APPPS1xP2X7Rko mice could be compensated by other pathways and notably, other purinergic receptors. Moreover, the finding that P2X7R deficiency did not affect IL-1β levels in APPPS1xP2X7Rko mice might explain the finding that P2X7R deficiency did not affect the phagocytic properties of microglia. Indeed, Ni et al. demonstrated that silencing P2X7R enhanced the microglia-dependent phagocytosis of Aβ1-42 in vitro, and this effect was reversed with IL-1β treatment [26]. Thus, the sustained IL-1β release in APPPS1xP2X7Rko mice may interfere with the hypothesized increase in phagocytosis associated with P2X7R deletion. In addition, P2X7R was shown to function as a scavenger receptor in absence of ATP and serum [47]. Among serum proteins, Gu et al. identified APP as an inhibitor of P2X7R-mediated phagocytosis [48]. Consequently, overexpression of APP and ATP release in APPPS1 mice would inhibit this P2X7R function, which could explain our observation that phagocytic activity was similar in APPPS1 and APPPS1xP2X7Rko mice.

We have shown that, in this Aβ model, P2X7R deficiency mainly affected the release of chemokines CCL3, CCL4, and CCL5. In particular, using cells from P2X7R-KO mice, we emphasized the specific role of P2X7R in Aβ-mediated CCL3 release from microglia and astrocytes. Interestingly, the expression of the chemokines CCL3, CCL4, and CCL5 and of their shared receptor CCR5 was increased in the brains of AD patients and in AD mice [7, 49–51]. Furthermore, the cognitive deficits and synaptic dysfunction induced by intracerebral injections of Aβ peptides were reduced in CCL3 and CCR5 knock-out mice [50]. Those data indicated that these chemokines may contribute to the development and progression of AD [38]. Indeed, chemokines can directly affect neuronal function [52]. Moreover, in support of this interpretation, CCL3 treatment of hippocampal slices and intracerebroventricular injections of CCL3 in WT mice impaired long-term synaptic plasticity and spatial memory abilities, and these effects were reversed with an antagonist of its receptor, CCR5 [53]. In addition, CCR5 overexpression in mice induced cognitive deficits, and conversely, CCR5 inhibition in the hippocampus improved memory [54]. Thus, P2X7R-dependent CCL3 release may directly alter neuronal functions, leading to synaptic and memory impairments. Notably, however, chemokines also contribute to numerous immune functions, such as immune cell recruitment in the CNS. Indeed, we showed a decrease in the number of CD8^+^ T cells in the hippocampus of APPPS1xP2X7Rko as compared to APPPS1 mice, which correlated with decreased CCL3 levels (Supplementary Figure 11k). These results were consistent with a previous report suggesting that CCL3 was involved in T cell infiltration in the brains of AD patients [55]. In addition, an increase in CCL3 levels was associated with T cell infiltration in the hippocampus of transgenic mice developing neurofibrillary tangles; moreover, in that model, T cell depletion improved cognitive impairments [51]. Those findings support the notion that P2X7R may contribute to neuropathological processes through CD8^+^ T cell recruitment, which is known to contribute to neurite damage, functional impairments, and neuronal apoptosis [56, 57]. Thus, P2X7R-dependent chemokines release could directly impair cognitive functions via CCR5 expression in neurons or through neuron/T cell interactions. Further studies inhibiting CCR5 are needed to either tease apart these two potential mechanisms or show that they work together in a single pathway that leads to AD progression (Supplementary Figure 12).

Taken together, these data showed that P2X7R deficiency reduced not only Aβ lesion but also improved synaptic plasticity. In recent years, several P2X7R antagonists were developed [15]; thus our results suggested that they may hold promise for the treatment of AD. P2X7R can have distinct biological functions in neurological disorders, depending on whether short-term or long-term signaling is involved [9, 15]. Based on our results, P2X7R appears to have different effects in acute AD model by activating microglia [10, 44] vs. chronic AD pathology by increasing chemokines production. Similarly, some inflammatory receptors, such as CX3CR1, have shown opposite effects on amyloid and Tau pathologies [58]. Considering that T-cell infiltration associated with CCL3 upregulation was found to play a role in detrimental processes involved in Tau pathology in a mouse model [51], inhibiting P2X7R might have a beneficial impact on Tau pathology. Other purinergic receptors play potential roles in neural and glial pathological processes involved in dementia. Indeed, a heterozygous deletion of the G protein-coupled P2Y2 receptor increases amyloid pathology and neurological deficits in an AD mouse model [59]. In contrast, blocking the A2A adenosine receptor reduced impairments in memory and synaptic trasnmission and reduced Tau phosphorylation and neuroinflammation in a mouse model of Tauopathy [57]. Thus, these preclinical studies have fueled interest in further explorations of interactions between purines, AD, and tauopathies.

In conclusion, our detailed analysis of P2X7R functions in a chronic AD mouse model uncovered an unexpected, pathological role of P2X7R in chemokine release in this neurodegenerative disease.

## Electronic supplementary material

Supplemental figures
