# Supplementary material for: New role of P2X7 receptor in an Alzheimer’s disease mouse model
Source: Mol Psychiatry. 2018 Jun 22;24(1):108–25. doi: 10.1038/s41380-018-0108-3 (PMC6756107; doi:10.1038/s41380-018-0108-3)
Supplement: Supplementary file 1 — Supplemental figures [file 41380_2018_108_MOESM1_ESM.pdf]

SUPPLEMENTARY FIGURES

Supplementary Figure 1.

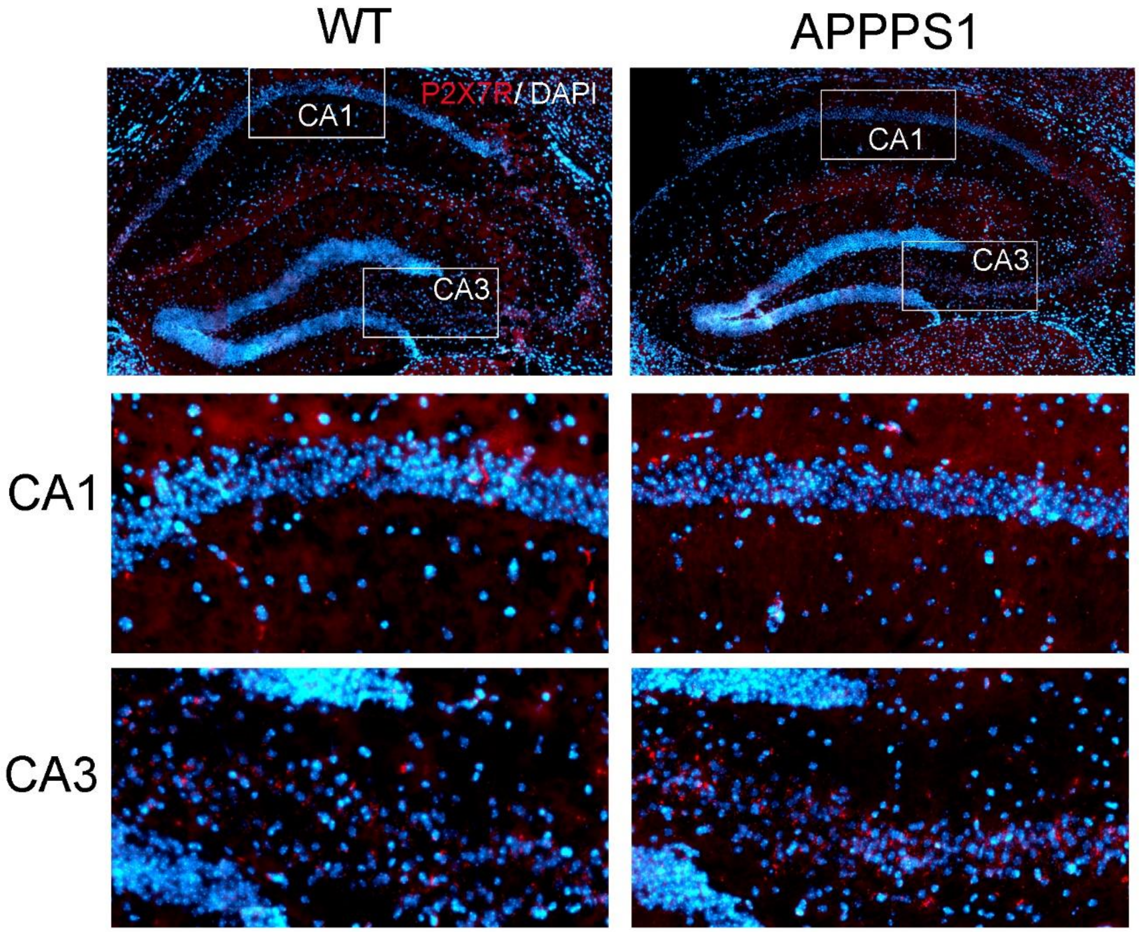

Supplementary Figure 1. P2X7R mRNA expression in the hippocampus of WT and APPPS1 mice. Brain slices from WT and APPPS1 mice were hybridized with RNAscope probes for P2X7R mRNA (red). Nuclei were counterstained with DAPI (blue).

Supplementary Figure 2.

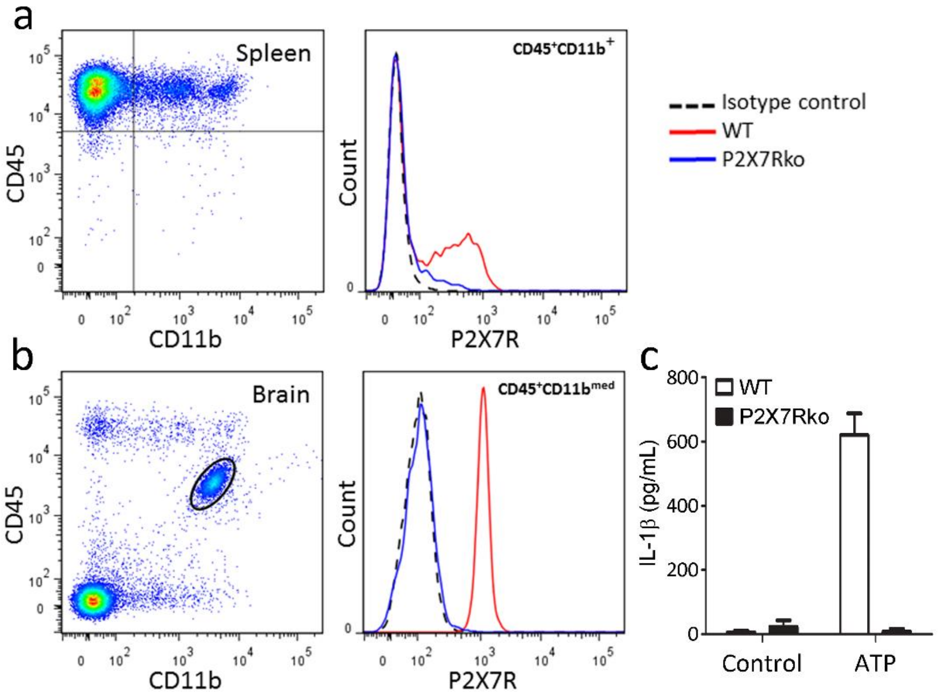

Supplementary Figure 2. **Macrophages and microglia from P2X7Rko mice do not express functional P2X7R**. Analyses of P2X7R surface expression on spleen macrophages and brain microglia by flow cytometry. **a** WT and P2X7Rko splenocytes were stained with anti-CD45 and anti-CD11b antibodies to identify macrophages (upper right quadrant) and labeled with P2X7R-specific or isotype control antibodies. **b** WT and P2X7Rko microglia were double-stained with anti-CD45 and anti-CD11b antibodies (gate) and labeled with P2X7R-specific or isotype control antibodies. **c** Analyses of IL-1β release from microglial cells after ATP stimulation. WT and P2X7Rko microglia were pretreated for 3 hours with LPS (100 ng / mL) and then stimulated or not with ATP (0.5 mM) for 30 minutes. IL-1β released in the supernatant was determined by ELISA.

Supplementary Figure 3

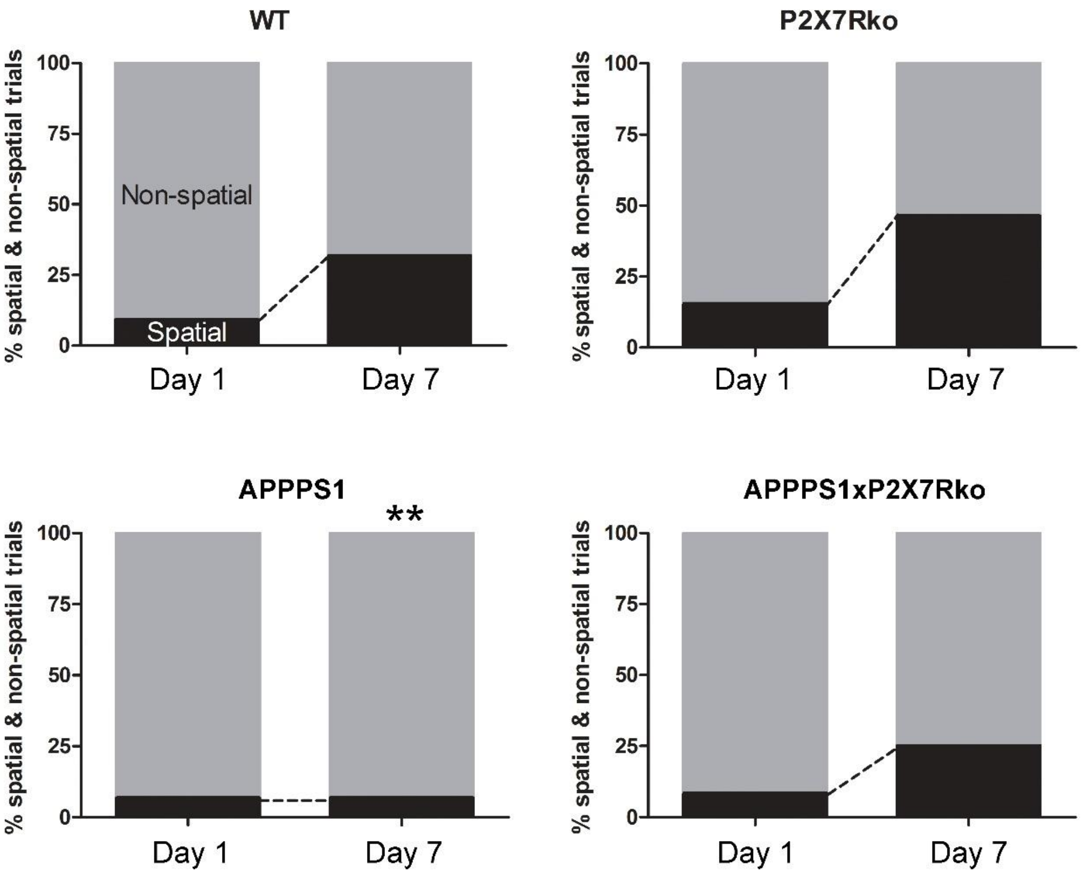

Supplementary Figure 3. **P2X7R deficiency improves the use of spatial strategies of APPPS1 mice.** Analysis of the strategies to reach the non-visible platform in the Morris Water Maze by 10-month-old male WT (*n* = 11), P2X7Rko (*n* = 13), APPPS1 (*n* = 11), and APPPS1xP2X7Rko (*n* = 6) mice. On day 7 versus day 1, WT mice performed a significantly higher number of spatial versus non-spatial trials compared to APPPS1 mice (Fisher's exact test: ** $P < 0.01$), while no difference was observed between WT and APPPS1xP2X7Rko mice ($P > 0.592$).

Supplementary Figure 4

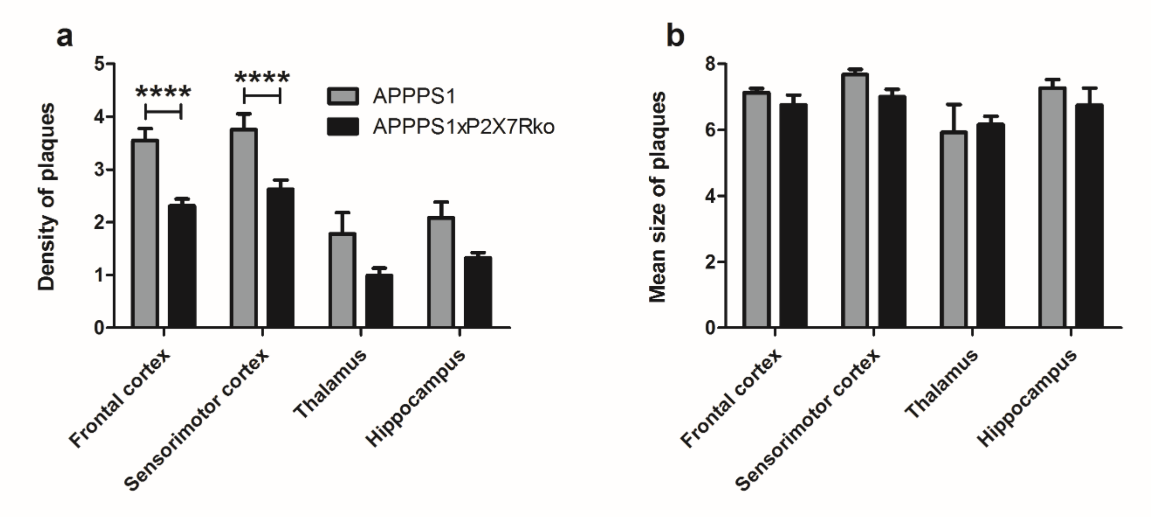

Supplementary Figure 4. **P2X7R deficiency decreases the number of Aβ plaques without affecting their mean size. a** Quantification of the number of Aβ plaques in different isocortical regions of 10-month-old APPPS1 (n = 5) and APPPS1xP2X7Rko (n = 4) mice. FC: frontal cortex, SMC: sensorimotor cortex, HC hippocampus, TL: thalamus. Two-way ANOVA with brain region and genotype as the principal factors, followed by Bonferroni's multiple comparison tests. **** P < 0.0001. **b** Quantification of the size of Aβ plaques in different isocortical regions of 10-month-old APPPS1 (n = 5) and APPPS1xP2X7Rko (n = 4) mice. FC: frontal cortex, SMC: sensorimotor cortex, HC hippocampus, TL: thalamus. Two-way ANOVA with brain region and genotype as the principal factors, followed by Bonferroni's multiple comparison tests.

Supplementary Figure 5

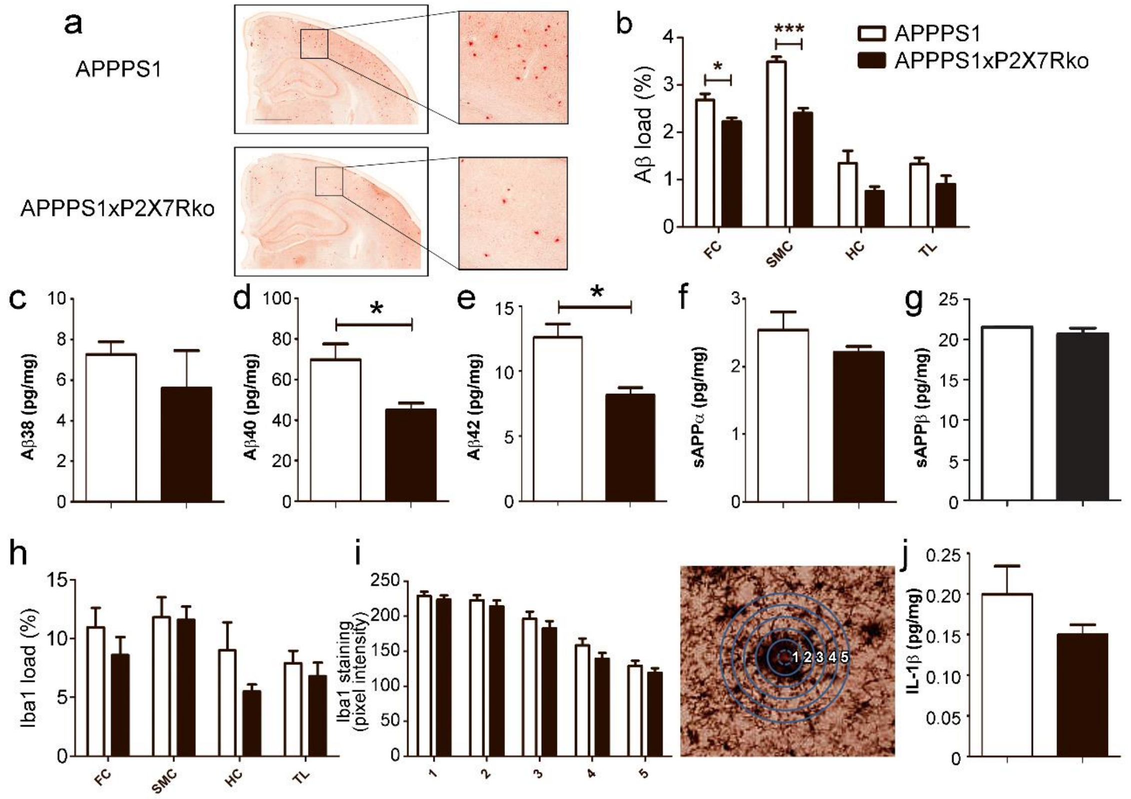

Supplementary Figure 5. P2X7R deficiency improves Aβ load in APPPS1 female mice without modifying microglia recruitment and IL-1β release. a Representative Congo red staining of brain slices from 8-month-old APPPS1 and APPPS1xP2X7Rko female mice. Scale bar: 1 mm. **b** Quantification of Aβ load in different isocortical regions of 8-month-old APPPS1 (n = 3) and APPPS1xP2X7Rko (n = 3) mice. FC: frontal cortex, SMC: sensorimotor cortex, HC hippocampus, TL: thalamus. Two-way ANOVA with brain region and genotype as the principal factors, followed by Bonferroni's multiple comparison tests. * $P < 0.05$, ***$P < 0.001$. **c-g** ELISA analyses of Aβ peptides, sAPPα and sAPPβ fragments in the brains of 8-month-old APPPS1 (n = 3) and APPPS1xP2X7Rko (n = 3) female mice. Student's t-test; *$P < 0.05$. **h** Analyses of Iba1 load in 8-month-old APPPS1 (n = 3) and APPPS1xP2X7Rko (n = 3) female mice and **i** periplaque microglial recruitment in 8-month-old APPPS1 (n = 3) and APPPS1xP2X7Rko (n = 3) mice. An overlay of 5 concentric circles centered on the Aβ core was applied (right panel) to determine Iba1 load as a function of the distance from the center of the plaque. At least 3 sections per mouse for Iba1 load and 10 plaques per mouse for microglial recruitment were used. Two-way ANOVA with brain region (**h**) or ring (**i**) and genotype as the principal factors, followed by Bonferroni's multiple comparison tests. **j** IL-1β protein expression was determined by multiplex ELISA on the brains of 8-month-old APPPS1 (n = 3) and APPPS1xP2X7Rko (n = 3) female mice. Student's t-test.

Supplementary Figure 6

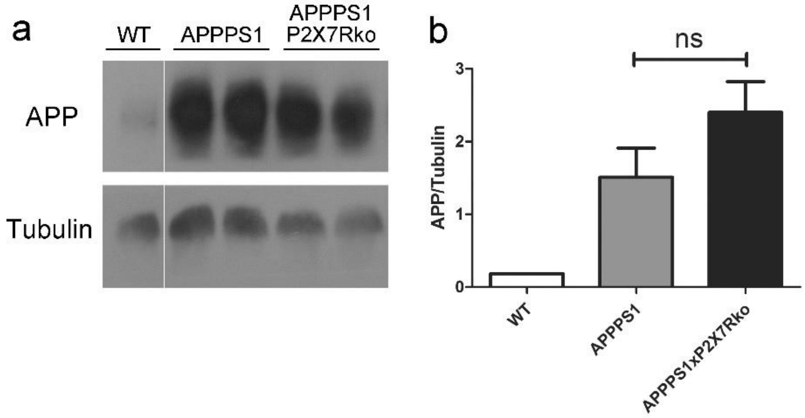

Supplementary Figure 6. Lack of P2X7R does not modify total APP level in the brain of APPPS1 mice. a Immunoblots were performed with mouse anti-APP and anti-tubulin antibodies on brain lysates from 10-month-old WT, APPPS1 and APPS1xP2X7Rko male mice. **b** Relative quantification of APP (APP/Tubulin) protein in the brain of WT, APPPS1 (n = 4) and APPPS1xP2X7Rko (n = 4) mice. Student's t- test; ns: not significant.

Supplementary Figure 7

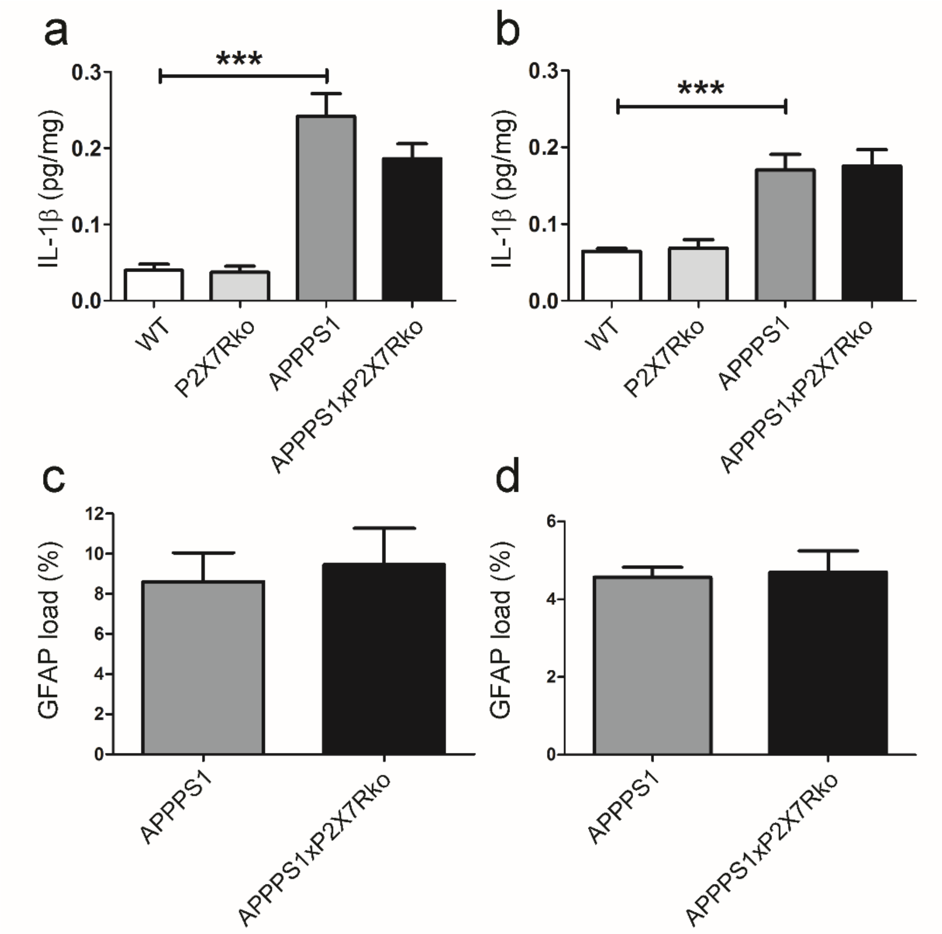

Supplementary Figure 7. P2X7R deficiency does not modify IL-1β release and GFAP load. IL-1β protein expression was determined in the brains of 12-month-old (**a**) WT (*n* = 6), P2X7Rko (*n* = 3), APPPS1 (*n* = 7) and APPPS1xP2X7Rko (*n* = 6) female mice and 15-month-old (**b**) WT (*n* = 9), P2X7Rko (*n* = 7), APPPS1 (*n* = 11) and APPPS1xP2X7Rko (*n* = 11) male mice by multiplex ELISA. One-way ANOVA followed by Tukey's multiple comparison test. ***P < 0.001. Analyses of GFAP load in the brains of 12-month-old (**c**) APPPS1 (n = 4) and APPPS1xP2X7Rko (n = 3) female mice and 15-month-old (**d**) APPPS1 (n = 10) and APPPS1xP2X7Rko (n = 11) male mice. Images were quantified for GFAP labeled area fraction (GFAP load (%)) by automated counting using ImageJ software. Briefly, images were normalized by subtracting background and an automatic thresholding (MaxEntropy) was applied.

Supplementary Figure 8

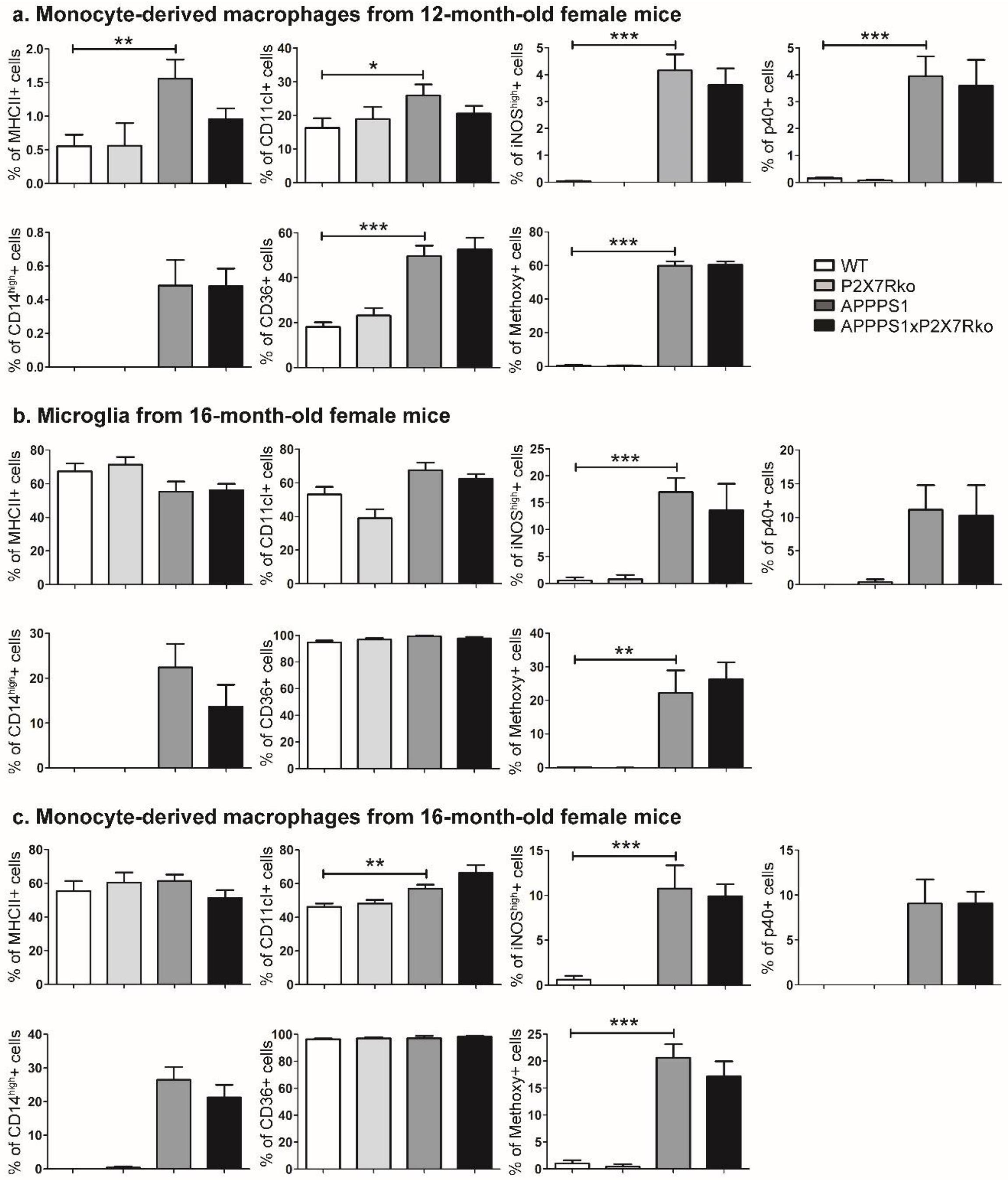

Supplementary Figure 8. P2X7R deficiency does not modify the phenotype of microglia and monocyte-derived macrophages. Percentage of CD11b^{+}CD45^{high} monocyte-derived macrophages (**a, c**) or CD11b^{+}CD45^{med} microglial cells (**b**) expressing MHCII, CD11c, iNOS, p40, CD14, CD36 and methoxy-X04 from 12-month-old (**a**) WT (n = 8), P2X7Rko (n = 7), APPPS1 (n = 5) and APPPS1xP2X7Rko (n = 6) female mice, and from 16-month-old (**b-c**) WT (n = 9), P2X7Rko (n = 5), APPPS1 (n = 7) and APPPS1xP2X7Rko (n = 10) female mice. One-way ANOVA followed by Tukey's multiple comparison test. *P < 0.05, **P < 0.01, ***P < 0.001.

Supplementary Figure 9

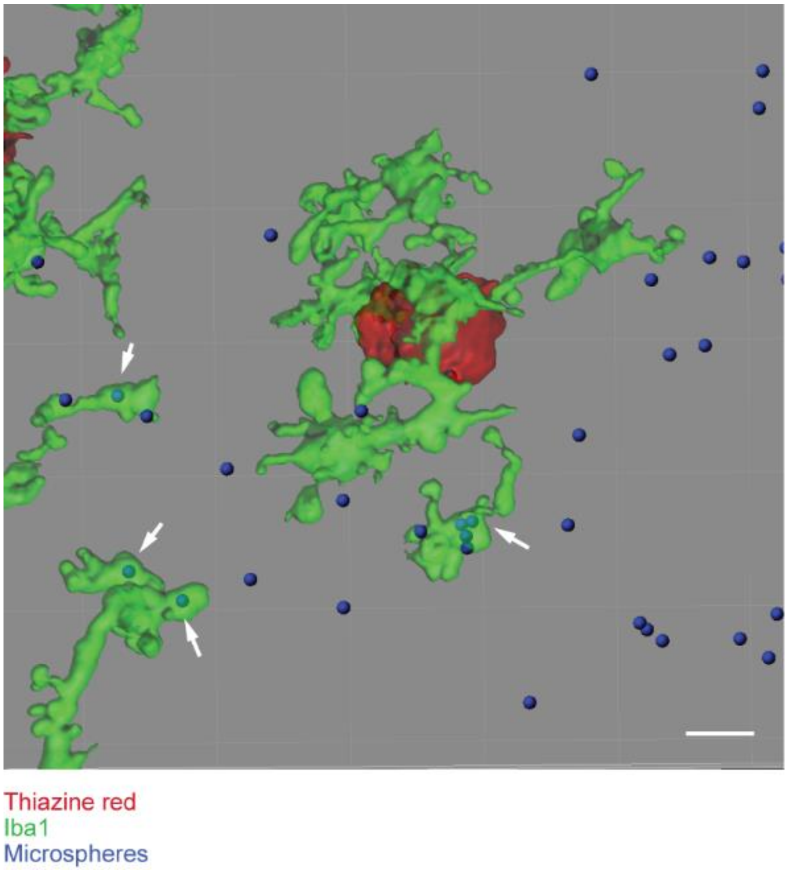

Supplementary Figure 9. Microglial cells engulfing fluorescent microparticles. Three-dimensionally reconstructed confocal images of microglial cells engulfing fluorescent microparticles with the software Imaris. Microglial cells are labelled with anti-Iba1 antibodies (in green) and amyloid plaques with thiazine red (in red) and fluorescent beads (in blue). The arrows indicate particles engulfed by microglial cells. Scale bar, 10 µm.

Supplementary Figure 10

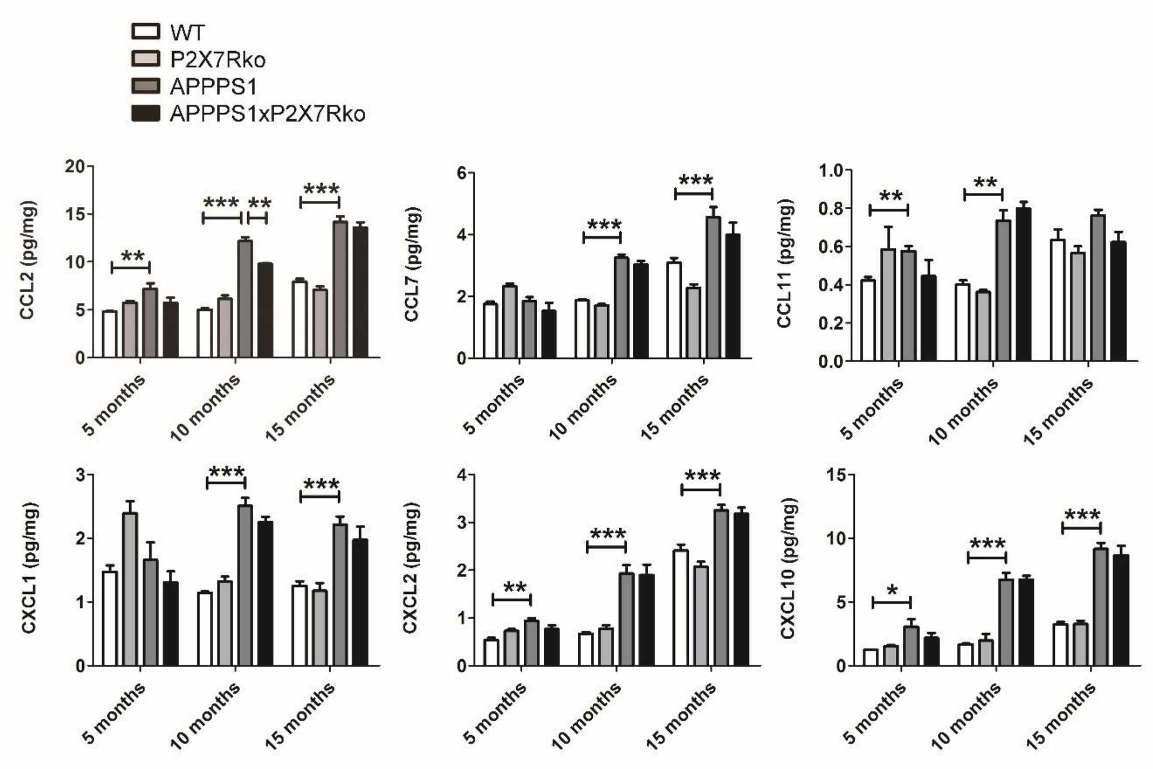

Supplementary Figure 10. Levels of cerebral chemokines in WT, P2X7Rko, APPPS1 and APPPS1xP2X7Rko mice. CCL2, CCL7, CCL11, CXCL1, CXCL2 and CXCL10 protein expression in the brains of 5-, 10- and 15-month-old male WT (*n* = 4-8), P2X7Rko (*n* = 3-6), APPPS1 (*n* = 4-11) and APPPS1xP2X7Rko (*n* = 4-10) mice was determined by multiplex ELISA. One-way ANOVA followed by Tukey's multiple comparison test. *P < 0.05, **P < 0.01, ***P < 0.001.

Supplementary Figure 11

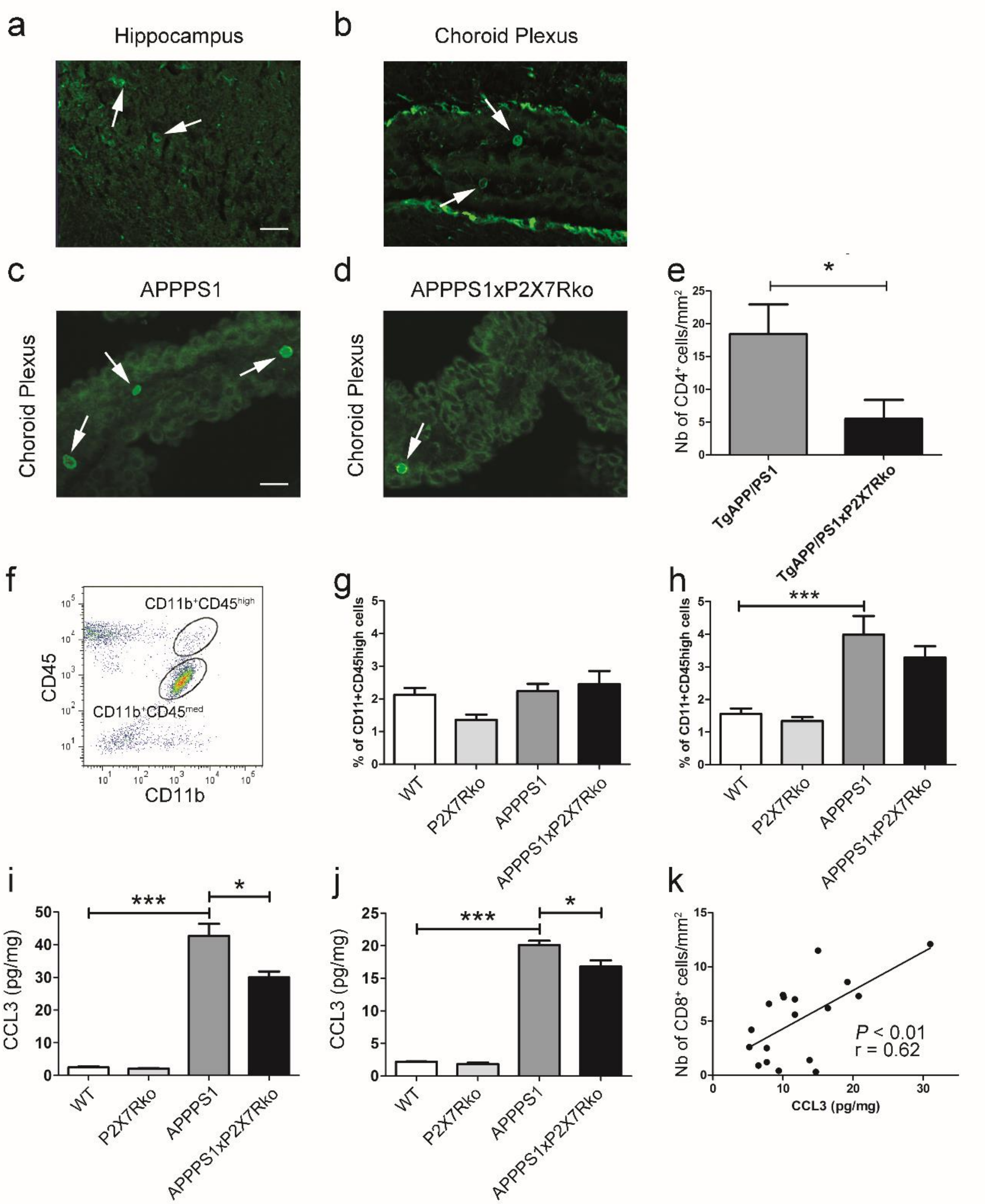

Supplementary Figure 11. P2X7R deficiency reduces the CCL-3 release associated with a decrease in CD4^{+} T-cell recruitment without changes in monocyte-derived macrophages (MDM) infiltration. CD3^{+} T cell labeling in the hippocampus (**a**) and choroid plexus (**b**) of 15-month-old APPPS1 mice. The arrows indicate CD3^{+} cells. Scale bar: 20 µm. CD4^{+} T cell labeling of brain sections from 15-month-old APPPS1 (**c**) and APPPS1xP2X7Rko mice (**d**). The arrows indicate CD4^{+} cells Scale bar: 20 µm. The number of CD4^{+} T cells was determined in the choroid plexus (**e**) of APPPS1 (n = 6) and

APPPS1xP2X7Rko mice (*n* = 5). Unpaired Student's t-test. *P < 0.05. FACS analysis of brain cells from 16-month-old APPPS1 mice stained with CD45 and CD11b antibodies. Gates shown in the dot plots illustrate the $CD11b^{+}CD45^{med}$ microglia population and the $CD11b^{+}CD45^{high}$ MDM population (**f**). Histograms indicate the percentage of $CD11b^{+}CD45^{high}$ cells of the total $CD11b^{+}$ cells from 12-month-old (**g**) WT (*n* = 8), P2X7Rko (*n* = 7), APPPS1 (*n* = 5) and APPPS1xP2X7Rko (*n* = 6) female mice and from 16-month-old (**h**) WT (*n* = 9), P2X7Rko (*n* = 5), APPPS1 (*n* = 7) and APPPS1xP2X7Rko (*n* = 10) female mice. CCL3 protein expression was determined in the brains of 12-month-old (**i**) WT (*n* = 7), P2X7Rko (*n* = 5), APPPS1 (*n* = 6) and APPPS1xP2X7Rko (*n* = 5) female mice and 18-month-old (**j**) WT (*n* = 3), P2X7Rko (*n* = 4), APPPS1 (*n* = 3) and APPPS1xP2X7Rko (*n* = 4) male mice by multiplex ELISA. One-way ANOVA followed by Tukey's multiple comparison test. *P < 0.05, **P < 0.01, ***P < 0.001. (**k**) CCL3 protein levels are positively correlated with hippocampal $CD8^{+}$ T-cell number. Correlations were generated using Pearson's correlation coefficient (*n* = 18). r = 0.62; **P > 0.01.

Supplementary Figure 12

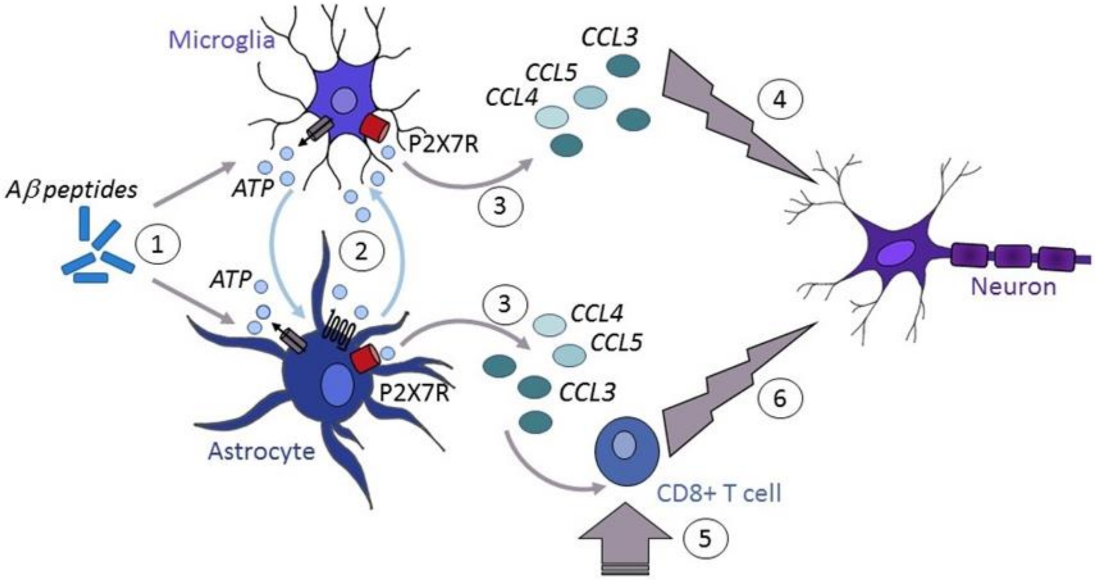

Supplementary Figure 12. Model of the roles of P2X7R in chronic Aβ disease. (**1**) Aβ peptides first induce ATP release from glial cells. (**2**) Activated astrocytes and microglia may in turn release ATP via purinergic receptors. (**3**) Increased levels of ATP activate P2X7R, leading to chemokine release from microglia and astrocytes. (**4**) Chemokine production might trigger direct neuronal impaired functions, damage neurites and (**5**) the recruitment of $CD8^+$ T cells, which also (**6**) contributes to neuronal toxicity.
